# Corrosion–Wear Synergistic Effect of Laser-Clad WC/Ni-Based Protective Coatings on P550 Nonmagnetic Drill Collar

**DOI:** 10.3390/ma19204354

**Published:** 2026-10-16

**Authors:** Lilan Liu, Yingkai Qin, Jiajia Li, Qiang Xu, Qifan Luo, Ziying Wu

**Affiliations:** School of Mechanical Engineering, Xi’an University of Technology, Xi’an 710048, China; 2240221189@stu.xaut.edu.cn (Y.Q.); 13155726787@163.com (J.L.); 2250220024@stu.xaut.edu.cn (Q.X.); 2250221227@stu.xaut.edu.cn (Q.L.); ziyingwu@163.com (Z.W.)

**Keywords:** nonmagnetic drill collars, laser cladding, protective coatings, corrosion–wear interaction

## Abstract

The coupled corrosion–wear damage of P550 nonmagnetic drill collars severely restricts their service reliability in downhole environments. However, the asymmetric synergistic interaction between corrosion and wear, and the underlying microscopic damage mechanism of P550 steel and its laser-clad protective coating under saturated brine drilling fluid conditions remain insufficiently understood. In this work, nonmagnetic WC/Ni-based protective coatings were fabricated on P550 substrates by laser cladding. Three groups of comparative tests, including pure corrosion, pure wear and corrosion–wear tests, were performed in saturated brine drilling fluid. The results show that the P550 substrate undergoes severe material degradation, while the WC/Ni-based coating exhibits superior corrosion and wear resistance benefiting from the skeleton-support and load-bearing effect of hard WC particles. Volumetric loss quantification demonstrates that the total material loss of the coating is markedly lower than that of the substrate, and the volume loss of both substrate and coating is dominated by pure mechanical wear and corrosion-accelerated wear, whereas pure corrosion and wear-accelerated corrosion make negligible contributions. Of the two coupled damage modes, corrosion-accelerated wear is much more pronounced than wear-accelerated corrosion, showing an obvious asymmetric synergistic damage in this system. Microscopic analysis indicates that surface microstructure deterioration and microdefect propagation are the primary drivers aggravating the coupled corrosion–wear damage. In summary, the laser-clad WC/Ni coating can substantially enhance the corrosion–wear resistance of P550 nonmagnetic drill collars. This work provides theoretical reference and engineering support for surface strengthening and service-life extension of nonmagnetic drill collars.

## 1. Introduction

Currently, oil and gas drilling operations are gradually expanding to deep and ultra-deep formations, leading to a significant increase in the extremity and complexity of downhole operating conditions and imposing more stringent requirements on the service performance of key components of drilling equipment [1]. As a core downhole tool in petroleum drilling, nonmagnetic drill collars can not only bear various mechanical loads during the operation, but also effectively eliminate or greatly reduce the interference of drill strings on the geomagnetic field, thereby providing a stable magnetic field environment for high-precision measurement while drilling (MWD) and logging while drilling (LWD) [2,3].

Owing to its high strength and excellent nonmagnetic properties, P550 stainless steel has become an important material for the manufacture of nonmagnetic drill collars [4,5]. However, under actual downhole service conditions, P550 stainless steel is simultaneously exposed to the corrosive action of drilling fluids and mechanical wear caused by drill-string rotation. Consequently, it is susceptible to coupled corrosion–wear failure, resulting in degradation of surface properties and reduced service reliability of nonmagnetic drill collars [6]. Applying a hard-alloy wear-resistant hardband coating to the critical surface regions of nonmagnetic drilling tools can effectively improve their wear and corrosion resistance, thereby extending their service life [7,8].

Laser cladding technology is an important approach for surface strengthening and protection of nonmagnetic drilling tools. In the field of laser cladding on stainless steel, Ji et al. [9] fabricated a cladding layer of the same material on P550 stainless steel, which is commonly used for nonmagnetic drill collars, and found that the corrosion resistance of the coating was improved compared with that of the substrate. Mao et al. [10] prepared a Ni-Cr-Mo corrosion-resistant alloy coating on the surface of P550 nonmagnetic stainless steel. The Ni-Cr-Mo cladding layer exhibited markedly superior pitting corrosion resistance to the substrate. However, low-speed laser cladding induced the formation of ferromagnetic phases in P550 nonmagnetic steel. Yang et al. [11] fabricated a high-hardness Ni60 coating and a lower-hardness WC-12Co/Ni25 composite coating on the W1813N nonmagnetic stainless steel by laser cladding. Tribological tests demonstrated that the WC-12Co/Ni25 composite coating exhibited better wear resistance. In addition, our research group [12] fabricated a nonmagnetic Ni-based coating with a high WC content on the surface of P550 nonmagnetic steel by laser cladding. A multiple-regression prediction model was employed to determine an optimal combination of process parameters that yielded an extremely low porosity in the WC-reinforced Ni-based coating. The resulting coating exhibited a hardness approximately 1.6 times that of the substrate.

The corrosion and wear resistance of laser-clad alloy coatings are critical indicators determining their service life and operational safety. At present, numerous scholars have conducted extensive research on the corrosion and wear behavior of laser-clad alloy coatings [13,14,15,16]. Luo et al. [17] prepared high-entropy alloy coatings with different Cr contents by laser cladding and confirmed a significant positive synergistic interaction between corrosion and wear. Corrosion was found to markedly aggravate wear failure, whereas an appropriate Cr content promoted the formation of a stable passive film. Guo et al. [18] reported that immersion in corrosive media continuously degraded the surface properties of the coating and increased the wear rate. The coupled damage of the coating in salt solution was governed by multiple mechanisms, including electrochemical corrosion, oxidative wear and three-body abrasive wear. Wang et al. [19] fabricated FeCrBSi medium-entropy alloy coatings via laser cladding. Experimental results verify a mutual promotion effect between corrosion and wear, and the wear damage induced by corrosion plays a dominant role in the coupled failure process. Sun et al. [20] demonstrated that the surface morphology and microstructure of Fe-based coatings can affect their corrosion–wear performance, and that an appropriate orientation of the working surface can effectively suppress coupled damage and improve service stability. Lu et al. [21] confirmed that the incorporation of WC hard particles into cladding coatings can optimize the coating microstructure, increase hardness, and improve the stability of the passive film, thereby significantly enhancing corrosion–wear resistance. In another study, Wu et al. [22] investigated the corrosion–wear behavior of hard coatings in sand-laden water and found that corrosion assisted in aggravating abrasive wear, although mechanical wear remained the dominant failure mode. In addition, Singh et al. [23] showed that laser-clad stainless-steel coatings can significantly reduce the corrosion rate of the substrate and form a stable protective surface layer, thereby effectively improving the corrosion–wear resistance of the substrate under oilfield service condition.

Existing studies demonstrate that laser-clad alloy coatings can markedly improve the service performance of metal components under coupled corrosion–wear conditions by tuning the microstructure, phase composition and increasing hardness. It is indicated that corrosion–wear is not a simple superposition of corrosion and mechanical wear. Instead, it represents a complex interfacial damage behavior with significant interaction effects. Nevertheless, certain limitations persist in current research. Most investigations separately examine either corrosion mechanisms or wear resistance, while the synergistic promotion effect between corrosion and wear lacks in-depth analysis. For strengthening coatings deployed on drilling tool surface, their corrosion–wear mechanisms have not been systematically studied. In particular, for nonmagnetic drill collars, the bidirectional interaction between corrosion and wear as well as the synergistic damage law in saturated brine drilling fluid remain insufficiently understood. The available research results cannot satisfy the protection requirements under such harsh service condition.

The existing works indicate that laser-clad alloy coatings can effectively improve the service performance of metallic components under corrosion–wear conditions by optimizing the surface microstructure, increasing material hardness, and enhancing corrosion resistance. However, current studies still have several limitations. Most investigations have focused on either individual corrosion mechanisms or wear resistance, whereas the interactions and synergistic effects between corrosion and wear have not yet been examined in sufficient detail. In addition, systematic studies on the corrosion–wear mechanisms of reinforced coatings operating in corrosive media, such as those encountered in oil and gas drilling equipment, remain limited. In particular, for nonmagnetic drill collars, the bidirectional interaction between corrosion and wear and the associated synergistic damage behavior in saturated brine drilling fluids have not been adequately clarified, making it difficult to fully meet the protection requirements imposed by complex service environments.

Therefore, to systematically elucidate the coupled corrosion–wear damage mechanisms of nonmagnetic drill collars and their protective coatings under complex downhole conditions, a nonmagnetic Ni-based WC composite coating was fabricated on a P550 stainless-steel substrate by laser cladding. Comparative experiments were conducted under pure corrosion, pure wear, and coupled corrosion–wear conditions in a saturated brine drilling fluid environment. Combining electrochemical testing, tribological performance analysis, and microstructure characterization, the macroscopic damage characteristics and microscopic evolution laws under different test conditions were systematically investigated. The interaction mechanisms between corrosion and wear were further clarified, together with the protection-enhancement and damage-suppression mechanisms of the Ni-based WC composite coatings. The results can provide a theoretical basis and technical support for optimizing surface modification processes for nonmagnetic drill collars, improving the coupled corrosion–wear resistance, and promoting the long-term safe service of nonmagnetic drill collars in downhole environments.

## 2. Experiments and Methods

### 2.1. Preparation of the Coating

**Figure 1:**
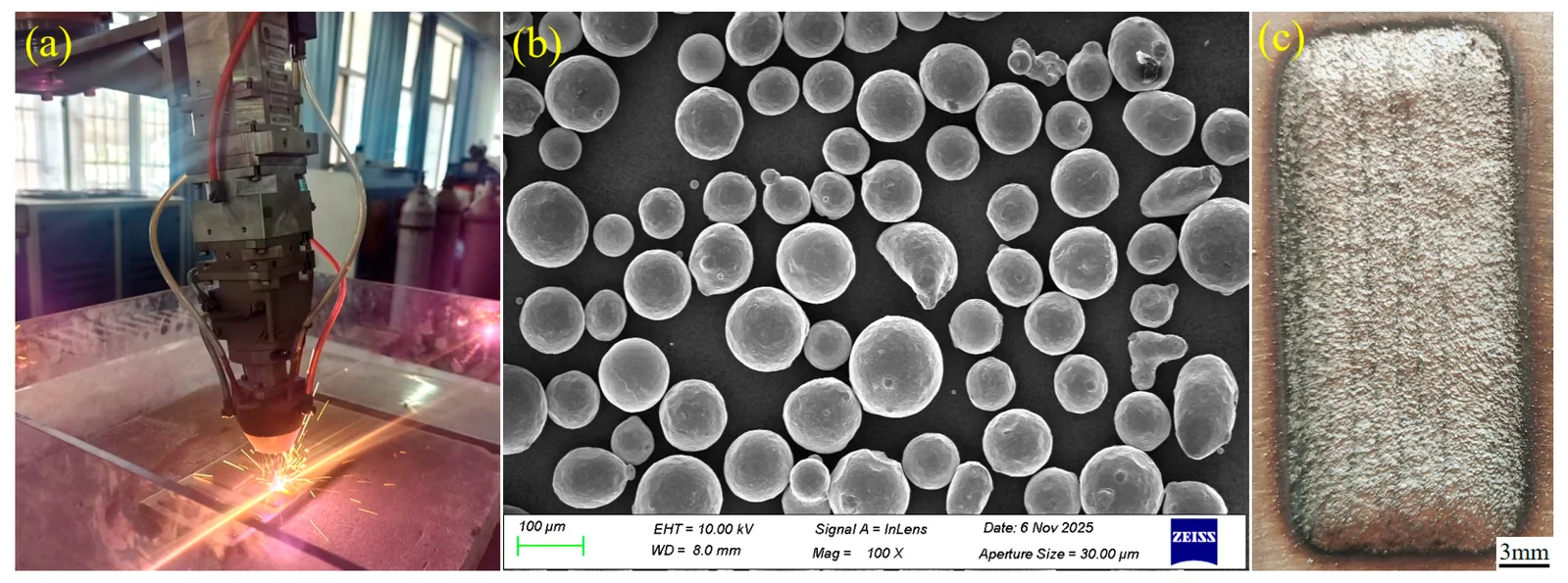
(**a**) Laser cladding experimental setup. (**b**) Powder. (**c**) Coating.

The laser cladding experiments were performed using a KUKA six-axis coordinated coaxial powder-feeding laser cladding system (Laserline Laser Technology Co., Ltd., Shanghai, China). The laser beam emitted by an LDF-3000-60 diode laser (Laserline Laser Technology Co., Ltd., Shanghai, China) produced a 3 mm spot on the substrate surface, as shown in Figure 1a. P550 nonmagnetic stainless steel was used as the substrate material, and the LC-WC60-1 nonmagnetic WC/Ni-based powder (Höganäs AB, Höganäs, Sweden) with a particle size ranging from 53 to 150 μm was used (Figure 1b). The chemical compositions of the P550 nonmagnetic steel and the nonmagnetic WC/Ni-based powder are listed in Table 1 and Table 2, respectively. Prior to laser cladding, the substrate surface was thoroughly ground with abrasive paper and subsequently cleaned with absolute ethanol to remove oil, contaminant particles and other surface impurities. The powder was dried before use. Based on the results of previous experimental studies [12], the laser cladding process parameters were set as follows: laser power of 1015 W, powder feeding speed of 0.6 r/min, overlap ratio of 40%, and scanning speed of 5.1 mm/s. The prepared coating is shown in Figure 1c.

### 2.2. Corrosion–Wear Tests

#### 2.2.1. Test System

**Figure 2:**
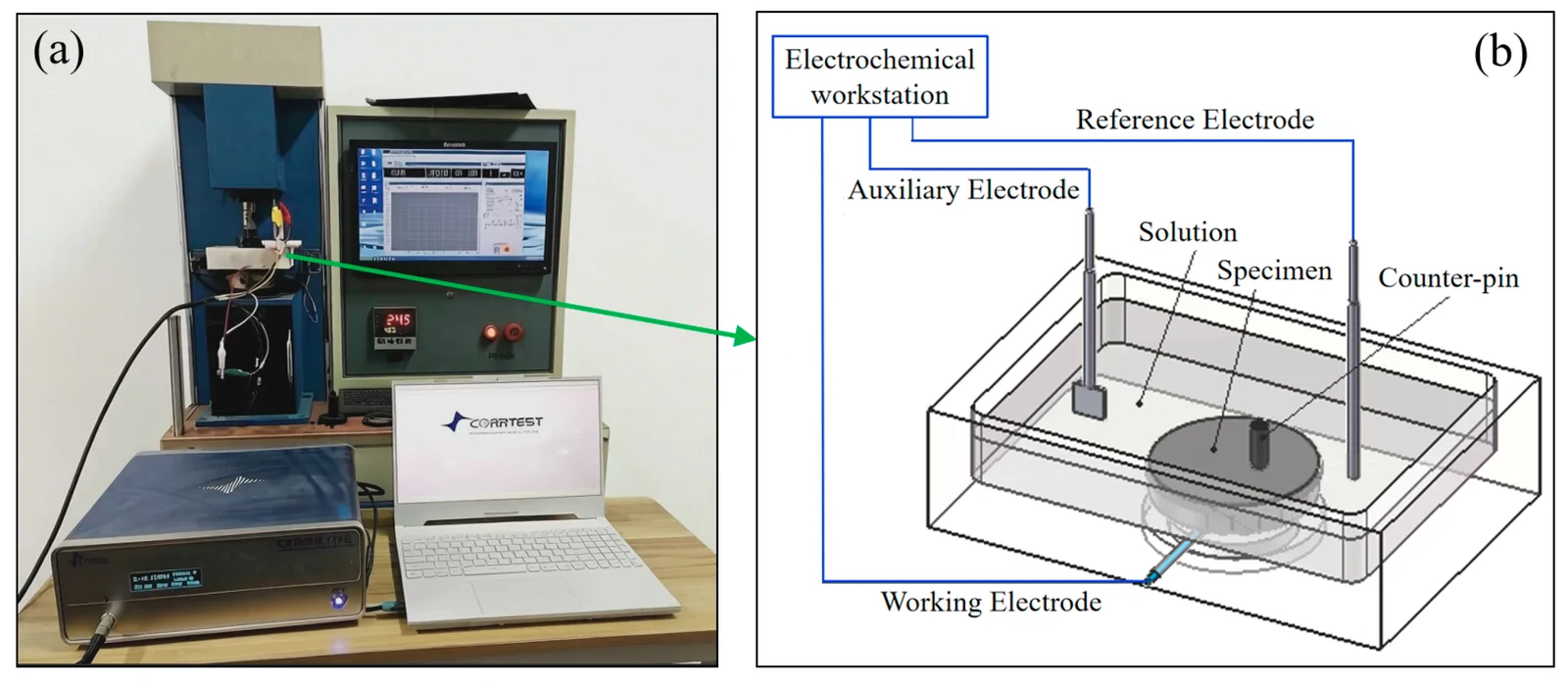
(**a**) Corrosion–wear test apparatus; (**b**) schematic diagram of corrosion–wear test.

A modified MMW-1A friction and wear tester coupled with an CS350M electrochemical workstation (Wuhan Corrtest Instruments Corp., Ltd., Wuhan, China) was used to establish a combined corrosion–wear test system for the P550 substrate and the WC/Ni-based coating, as shown in Figure 2. Both the substrate and coating specimens had dimensions of Φ50 mm × 10 mm, and all specimens were sequentially polished with 400#, 800#, 1200#, and 2000# abrasive papers until the surface is smooth and free of scratches. A standard three-electrode system was adopted for all electrochemical measurements, wherein the prepared coating/substrate specimen served as the working electrode, a platinum sheet was used as the counter electrode, and a silver (Ag) electrode was applied as the reference electrode. The corrosive medium used was a saturated brine drilling fluid with a pH of 9. The drilling fluid was prepared by using distilled water as the base fluid, slowly adding NaCl powder under continuous stirring until saturation, and then adding 2 wt.% bentonite and 2 wt.% fluid-loss additive relative to the mass of the base fluid. The mixture was stirred until a homogeneous suspension without visible sediment was obtained. The pH of the drilling fluid was adjusted to 9 by dropwise addition of HCl or NaOH solution.

#### 2.2.2. Test Scheme

Three groups of controlled experiments were designed in this study, with three parallel specimens set for each group to guarantee data reliability. All electrochemical and tribological tests were conducted at room temperature (25 ± 3 °C) in saturated brine drilling fluid. Before and after testing, the specimens were immersed in alcohol and ultrasonically cleaned to remove surface contaminants and adherent residues, and then polished, cleaned and dried to a constant mass. The detailed experimental scheme is presented as follows:

Test 1: Pure corrosion test under static conditions. Electrochemical corrosion test was carried out under static condition, where the specimen was only subjected to the corrosion effect of drilling fluid, so as to quantitatively evaluate the independent influence of corrosion. Specifically, the specimen was immersed in the drilling fluid and kept standing for 30 min until the open circuit potential (OCP) stabilized. Subsequently, potentiodynamic polarization test was performed with a scanning range from OCP −1.2 V to 0.6 V and a scanning rate of 1 mV/s.

Test 2: Pure wear test under cathodic protection. Under cathodic protection conditions, the electrochemical corrosion effect of the drilling fluid was suppressed so that the contribution of mechanical wear could be quantified independently. Specifically, the specimen was immersed in the drilling fluid and held for 30 min until the OCP stabilized. A cathodic protection potential of OCP—1.0 V was then applied to the specimen, after which the friction test was initiated. A commercial Al_2_O_3_ ceramic ball was used as the counter body. The Al_2_O_3_ ball had a diameter of 6 mm, a Vickers hardness of 1800 ± 35 HV_0.5_, and surface roughness *R*_a_ below 0.02 μm. The tribological test was performed under a ball-on-disk point contact configuration. The tribological test parameters were as follows: normal load of 50 N, disk rotational speed of 100 r/min, total test duration of 30 min, and friction circle radius of 25 mm.

Test 3: Corrosion–wear test under dynamic polarization. Sliding friction tests were performed simultaneously with electrochemical corrosion in drilling fluid. Under dynamic polarization condition, the material was subjected to the combined effects of electrochemical corrosion, mechanical wear, and their synergistic interaction. In detail, the specimen was immersed in drilling fluid and stabilized for 30 min to achieve a steady OCP. Subsequently, potentiodynamic polarization tests and friction–wear tests were conducted synchronously. The potentiodynamic polarization scanning range was set from OCP−1.2 V to 0.6 V at a scanning rate of 1 mV/s. The friction test conditions were consistent with those in Test 2.

#### 2.2.3. Characterization

The coating microstructure was observed using an inverted metallurgical microscope (LWD300LCS, Cewei Optoelectronic Technology Co., Ltd, Xi’an, China). The coating cross-sections were cut by electrical discharge machining (EDM) and were ground stepwise using abrasive papers and then polished to a mirror finish. The polished surface was etched in diluted aqua regia solution (concentrated hydrochloric acid: concentrated nitric acid = 3:1 by volume), and the cross-sectional microstructure was observed. In addition, the phase composition was analyzed via X-ray diffraction (XRD, DX-2700BH, Haoyuan Instrument Co., Ltd, Dandong, China). The longitude microhardness of the coating was measured by a Vickers hardness tester (HV-1000STA, Cewei Optoelectronic Technology Co., Ltd, Xi’an, China). The press load was 0.5 kgf and was held for 15 s. At each measured position, the hardness of three points were measured and took the average to reduce test errors.

Tribological characterization was performed to quantitatively evaluate the friction and wear performance. A laser scanning confocal microscope (Zeiss LSM880 Airyscan, ZEISS, Oberkochen, Germany) was used to characterize the two-dimensional and three-dimensional surface morphologies of the specimens, as well as to measure the cross-sectional area of worn tracks for wear volume calculation. In addition, the wear surface morphologies were observed using a scanning electron microscope (SEM, TESCAN MIRA4, TESCAN Trading Co., Ltd., Shanghai, China), and the surface elemental distribution of the coating was analyzed using an energy-dispersive spectrometer (EDS, Xplore30, TESCAN Trading Co., Ltd., Shanghai, China).

The material wear volume loss was calculated using the following equation:

*V* = 2*πrA* (1)

where *V* is the wear volume loss, *A* is the cross-sectional area of the wear track, and *r* is the friction circle radius.

According to Faraday’s law, the electrochemical corrosion volume loss was calculated by

(2) $$V^{\prime }=\frac{I_{\mathrm{coor}}A_{c}tM_{e}}{\mathrm{nF}\rho }$$

where *V*′ is the corrosion volume loss, *I*_corr_ is the corrosion current density, *A*_c_ is the exposed electrode area of the specimen, *t* is the test duration, *M*_e_ is the equivalent molar mass of the material, n is the valence state of the element, F is the Faraday constant, and ρ is the density of the material. For the equivalent molar mass *M*_e_ of the specimens, the mass fraction weighted-average method is adopted. The density ρ of specimens was obtained via experimental measurement. The electron transfer number n is approximately determined according to the anodic oxidation reaction of the main dissolved metallic element during the corrosion process. The dissolved metallic elements were selected as follows: Fe for P550 steel and Ni for the WC/Ni-based coating because WC is hardly corroded.

## 3. Results and Discussion

### 3.1. Characteristics of WC/Ni-Based Coating

**Figure 3:**
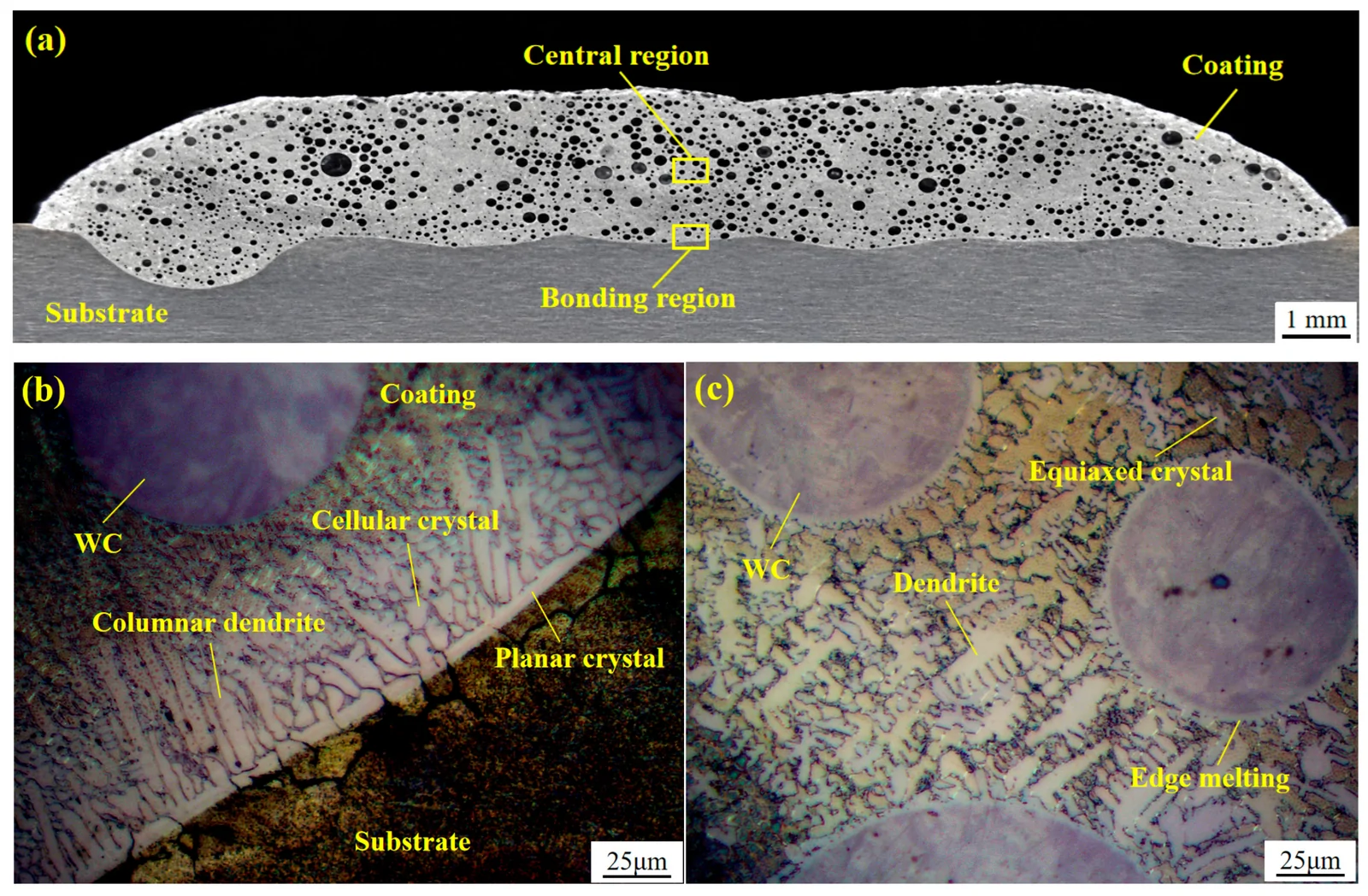
(**a**) Cross-section of the coating. (**b**) Microstructure of bonding region. (**c**) Microstructure of central region.

Figure 3 shows the cross-sectional view of the WC/Ni-based coating laser-clad on the P550 substrate and its metallographic microstructures (etched with 8% nitric acid solution) of the bonding region and the central region. In Figure 3a, it is illustrated that most of the WC particles retain their spherical morphology and embed uniformly in the Ni-based binder phase. In Figure 3b, a thin planar crystal layer forms at the bonding interface between the coating and substrate, indicating a dense metallurgical bonding. This is because a large temperature gradient and low constitutional undercooling at the interface facilitate the growth of planar thin crystals. Above this thin crystalline layer, columnar dendrites perpendicular to the bonding interface appear. Owing to the high cooling rate near the substrate, grains preferentially grow via reverse epitaxial growth during nucleation. Under a negative temperature gradient, the microstructure grows in a dendritic form. In the central region of the coating (Figure 3c), the microstructure is mainly composed of dendrites and equiaxed crystals. It can be seen that the WC particles considerably impede the oriented growth of crystals, and so short and thick dendritic crystals appeared. In addition, under laser irradiation, partial melting occurs at the edges of the WC particles.

**Figure 4:**
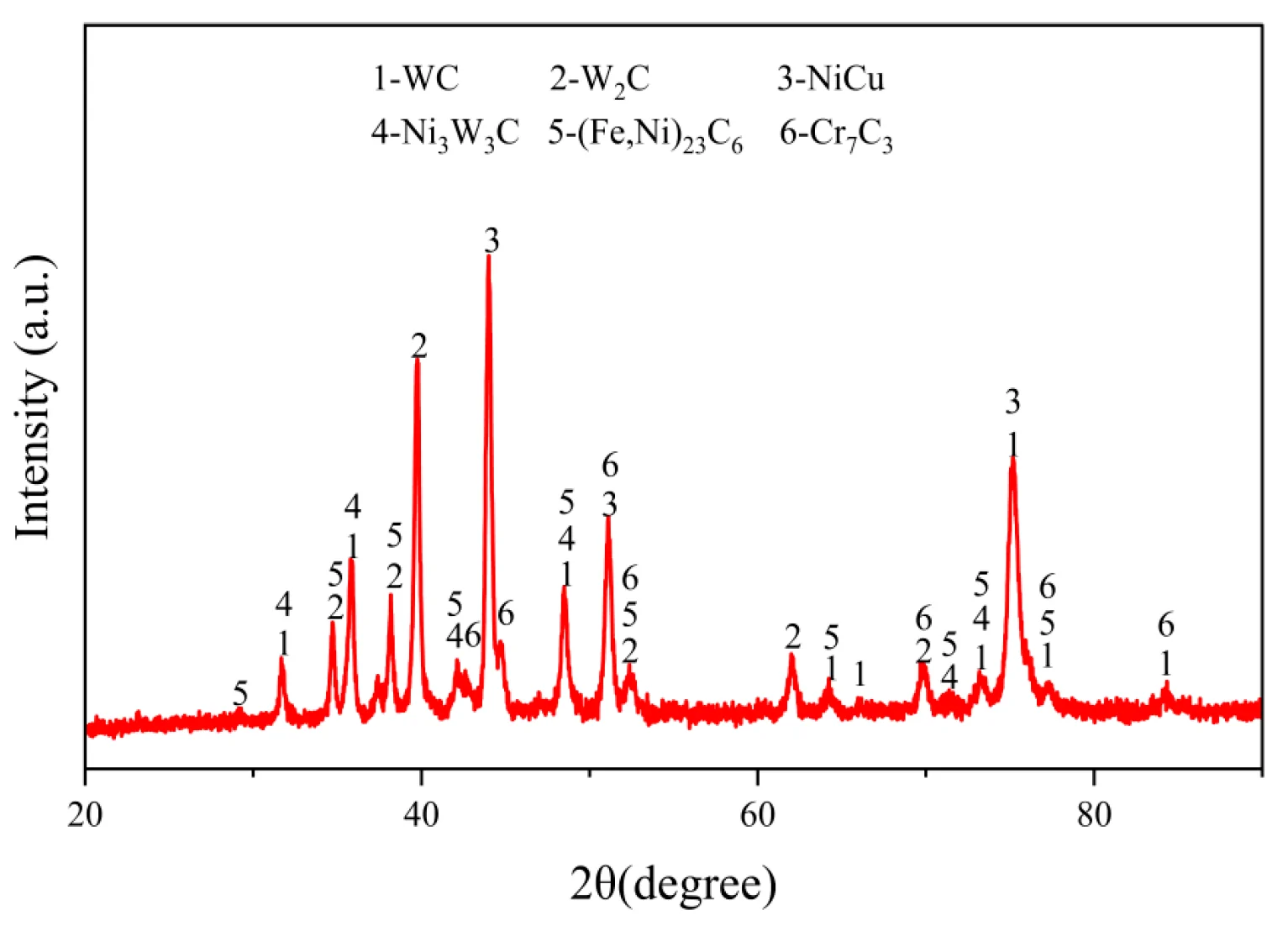
XRD pattern of WC/Ni-based coating.

The edge melting of WC particles can release W and C elements to diffuse into the molten pool, while Ni, Cr, and Fe in the molten pool will also infiltrate into their surface layer. The XRD analysis of the central region of the coating is shown in Figure 4. The main phases identified in the coating are WC, W_2_C, NiCu, Ni_3_W_3_C, (Fe,Ni)_23_C_6_, and Cr_7_C_3_. The characteristic diffraction peaks of WC are sharp and relatively intense, corresponding to the WC particles retained in the coating. The binder phase is dominated by a Ni-Cu solid solution, which exhibits pronounced characteristic diffraction peaks and constitutes the continuously distributed matrix phase of the coating. This phase is formed through mutual dissolution of Ni and Cu at elevated temperatures. In addition, the characteristic diffraction peak intensity of W_2_C is slightly lower than that of WC, which is generated by the decarburization decomposition of WC that underwent edge dissolution. The basic reaction is as follows: W_2_C is generated by decarburization and decomposition of WC during partial edge dissolution, and the basic reaction is

2WC → W_2_C + C (3)

At the same time, Ni_3_W_3_C, (Fe,Ni)_23_C_6_, and Cr_7_C_3_ are secondary phases. Their diffraction peaks are relatively weak, indicating that these phases are products of elemental diffusion and high-temperature reactions during the laser cladding process. Ni_3_W_3_C is formed through the reaction of W and C released from WC with Ni. (Fe,Ni)_23_C_6_ originates from the reaction of Fe in the feedstock powder with Ni and C, whereas Cr_7_C_3_ is formed by the reaction between trace amounts of Cr in the powder and free C, i.e.,

3Ni + 3W + C → Ni_3_W_3_C (4)

7Cr + 3C → Cr_7_C_3_ (5)

23(Fe,Ni) + 6C → (Fe,Ni)_23_C_6_ (6)

**Figure 5:**
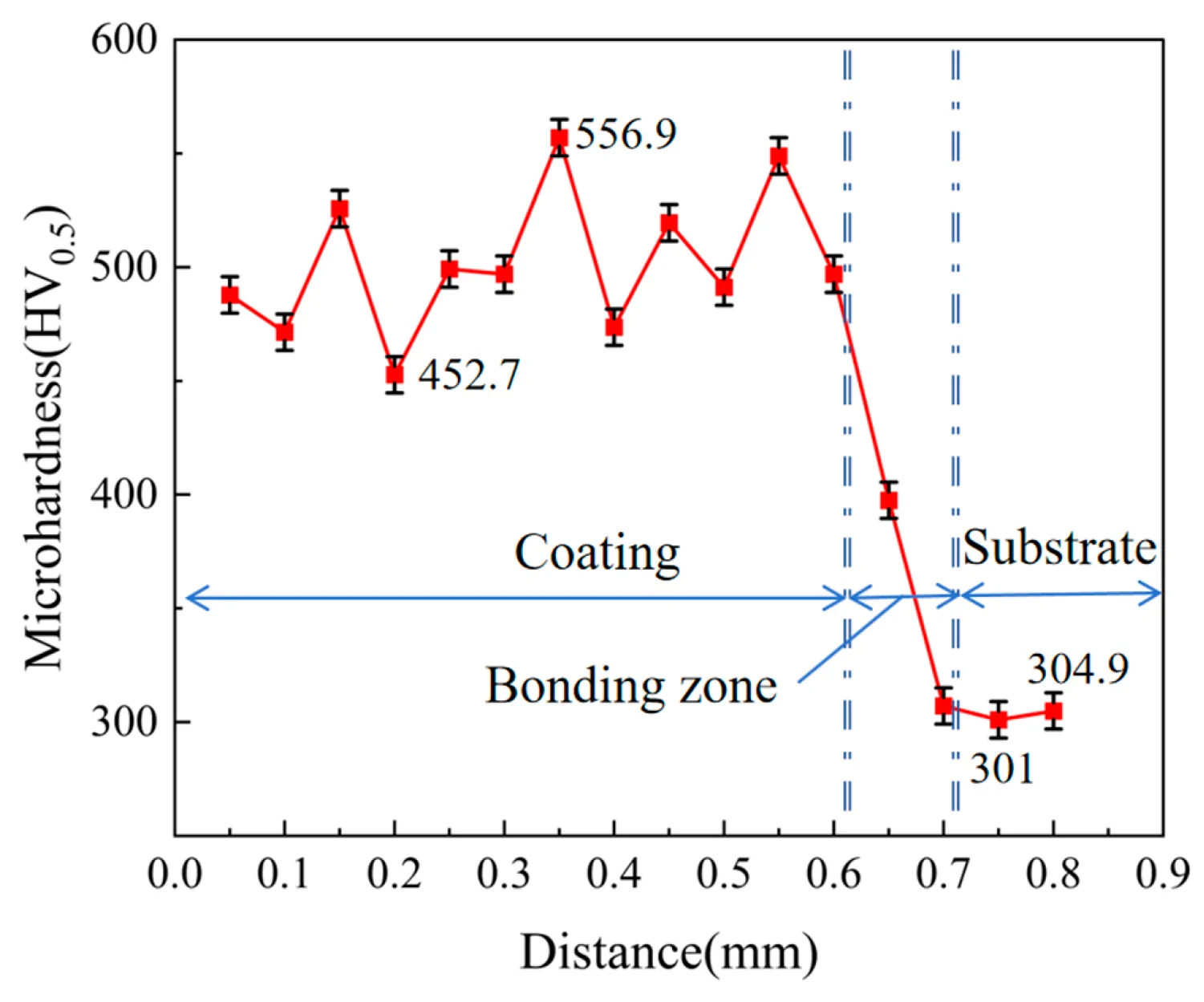
Microhardness of WC/Ni-based coating.

Figure 5 shows the longitudinal microhardness of the coating. It can be seen that the measured hardness of the coating fluctuates in the range of 452.7–556.9 HV_0.5_, with an average value of 489 ± 22 HV_0.5_. The average microhardness of the substrate is approximately 303 ± 15 HV_0.5_. The microhardness at the bonding zone decreases significantly due to the infiltration of P550 substrate material. The hardness of the coating is about 1.6 times that of the P550 substrate. This is because the WC ceramic particles, together with the newly formed hard phases such as W_2_C, (Fe,Ni)_23_C_6_ and Cr_7_C_3_, contribute to dispersion strengthening inside the coating, thus improving its hardness and wear resistance.

### 3.2. Pure Corrosion Under Static Polarization Condition

**Figure 6:**
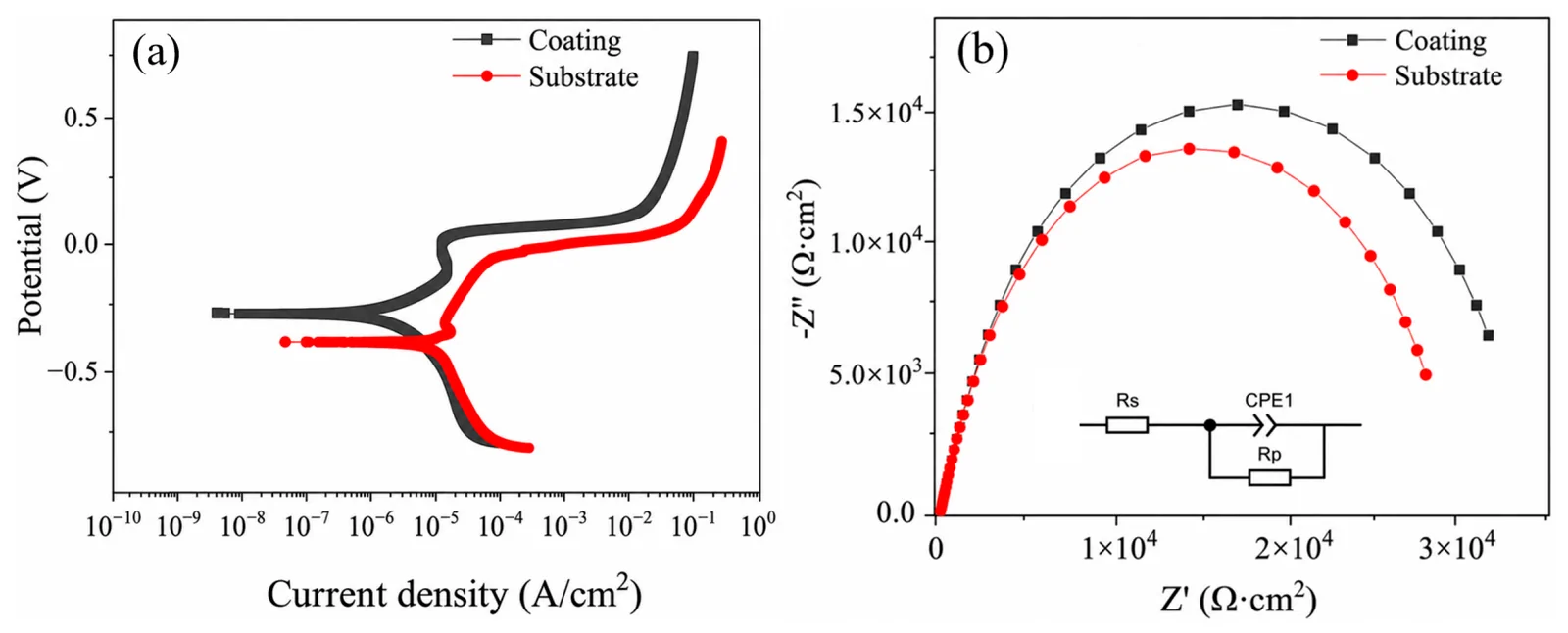
Results of pure corrosion: (**a**) Polarization curves. (**b**) Electrochemical impedance spectra.

To quantitatively evaluate the material loss of P550 substrate and the WC/Ni-based coating caused solely by corrosion, electrochemical corrosion tests were conducted on the specimens. Figure 6 shows the polarization curves and electrochemical impedance spectra (EIS) of one of the specimens in drilling fluid. The corrosion potential *E*_corr_ and corrosion current density *I*_corr_ were obtained by Tafel extrapolation. The EIS were fitted via CS Analysis using the nonlinear least-squares method. According to the single depressed capacitive semicircle observed in Nyquist plots, the equivalent circuit of *R*_s_(*R*_p_¦¦*CPE*_1_) was adopted. In this circuit, *R*_s_ is the solution resistance, *R*_p_ is the polarization resistance, and *CPE*_1_ is a constant phase element instead of an ideal capacitor to describe the non-ideal capacitive response caused by surface roughness and inhomogeneity. The *CPE*_1_ exponent *n*_1_ characterizes the deviation extent from the ideal capacitor.

The obtained electrochemical parameters are listed in Table 3. As indicated by the polarization curves and electrochemical parameters, the *I*_corr_ of the coating (2.68 ± 0.13 × 10^−6^ A/cm^2^) is significantly lower than that of the substrate (1.43 ± 0.08 × 10^−5^ A/cm^2^), indicating a lower corrosion rate of the coating. Meanwhile, the *E*_corr_ of the coating is −0.266 ± 0.010 V, which is markedly more positive than that of the substrate (−0.373 ± 0.013 V), suggesting a lower thermodynamic tendency toward corrosion. In addition, the *R*_p_ of the coating is 35,084 ± 1460 Ω·cm^2^, higher than the 32,846 ± 1168 Ω·cm^2^ of the substrate. The *CPE*_1_ of the coating is 5.71 ± 0.23 × 10^−5^, lower than the 5.85 ± 0.21 × 10^−5^ of the substrate. A lower passive-film capacitance generally corresponds to a denser passive film and a stronger barrier effect, indicating that the passive film formed on the coating surface is more compact than that formed on the substrate. The obtained *n*_1_ values (0.87 ± 0.13 for coating and 0.83 ± 0.13 for substrate) are less than 1, which is attributed to surface roughness and microstructural inhomogeneity of the tested specimens. Meanwhile the coating surface is relatively more homogeneous. All χ^2^ values were less than 1 × 10^−3^, indicating reliable fitting quality. These results further confirm that the coating exhibits a substantially lower corrosion rate than the substrate.

**Figure 7:**
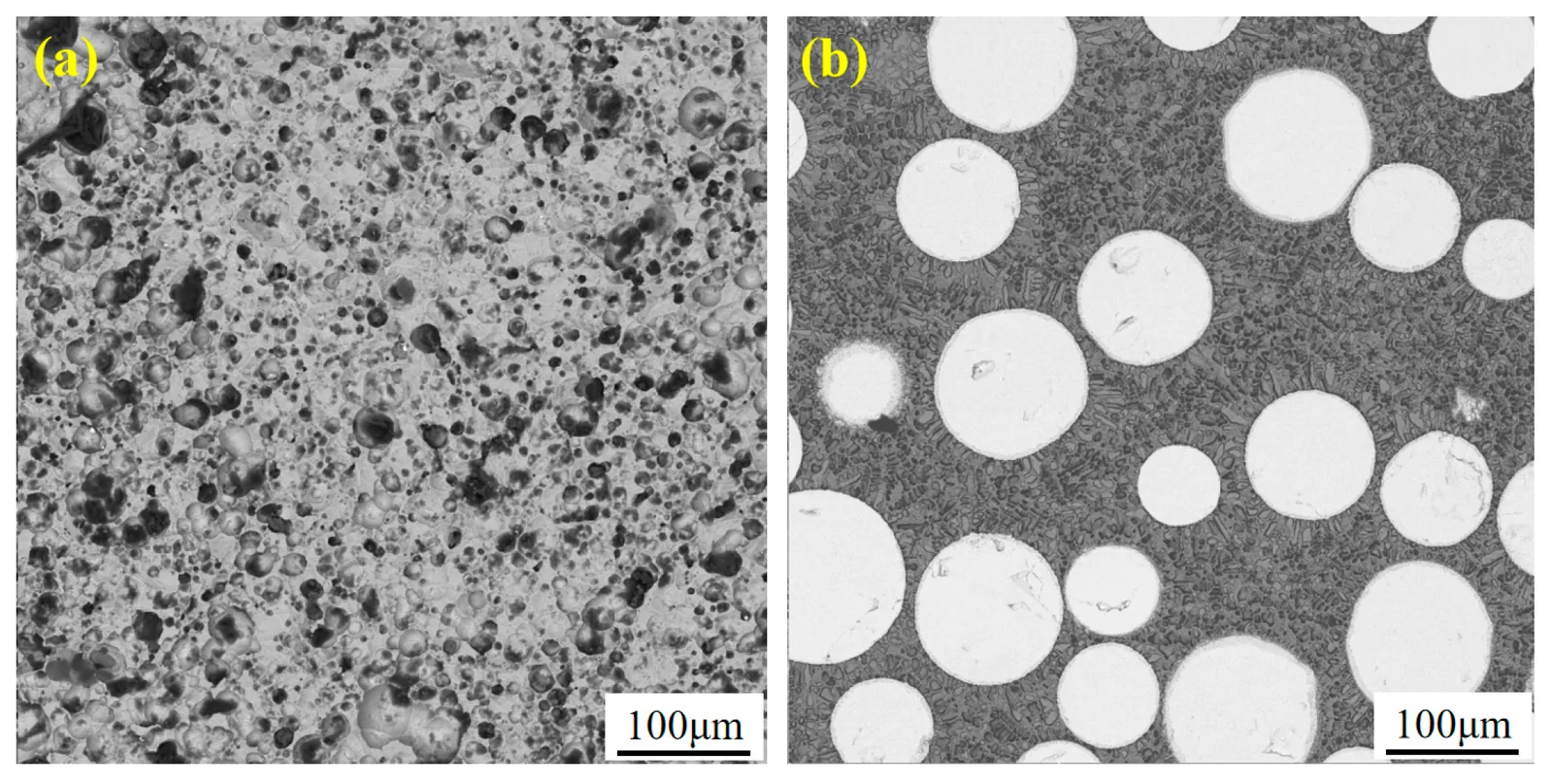
Corrosion morphologies of specimens: (**a**) P550 substrate; (**b**) WC/Ni-based coating.

Figure 7 shows the corrosion morphologies of the P550 substrate and the WC/Ni-based coating after static polarization in drilling fluid with pH = 9. The substrate exhibits uniform corrosion, where the surface undergoes general dissolution with numerous pits formed. In contrast, the coating exhibits selective corrosion with markedly lower corrosion severity. The WC hard particles within the coating remain largely intact, demonstrating that the WC phase possesses high chemical stability in Cl^−^ and OH^−^ containing media and barely participates in the primary corrosion reaction. The WC particles construct a stable corrosion-resistant skeleton in the binder phase. Corrosion preferentially occurs in the Ni-Cu binder, which presents a fine honeycomb-like morphology.

P550 nonmagnetic steel is mainly composed of Fe with relatively high contents of Cr and Mn. In weakly alkaline saturated brine drilling fluid containing high concentrations of Cl^−^, its corrosion process is jointly controlled by the anodic dissolution of metal and the cathodic reduction reaction of dissolved oxygen.

In the anodic regions, active constituents such as Fe, Cr and Mn are first dissolved to produce Fe^2+^, Mn^2+^, and Cr^3+^. Among them, Fe^2+^ reacts with OH^−^ in the medium to form Fe(OH)_2_, which is subsequently oxidized by dissolved oxygen to form corrosion products such as FeOOH:

Fe^2+^ + 2OH^−^ → Fe(OH)_2_↓ (7)

4Fe(OH)_2_ + O_2_ → 4FeOOH + 2H_2_O (8)

Meanwhile, Cr^3+^ can combine with OH^−^ to form a certain amount of passive film:

Cr^3+^ + 3OH^−^ → Cr(OH)_3_↓ (9)

2Cr(OH)_3_ → Cr_2_O_3_ + 3H_2_O (10)

However, in saturated brine drilling fluid, large amounts of Cl^−^ can preferentially adsorb onto and migrate toward defects, pores, and locally discontinuous regions of the passive film, where they undergo complexation reactions with dissolved metal ions such as Fe^2+^, Cr^3+^ and Mn^2+^: M^n+^ + mCl^−^ ⇌ [MCl_m_]^(n−m)^ (M denotes the metal element, n is its valence state and m is the coordination number). This reaction weakens the chemical bonds within the passive film and enhances the dissolution and transport of local corrosion products, thereby compromising the integrity of the passive film and inhibiting its regeneration. Therefore, although P550 can form a certain degree of surface passivation owing to its Cr content, the passive film is unable to maintain long-term integrity and stability under continuous attack by high concentrations of Cl^−^. As a result, extensive and sustained anodic dissolution occurs over the material surface, leading to the formation of numerous corrosion pits and ultimately producing the widespread corrosion morphology.

For the WC/Ni-based coating, the Ni-Cu binder phase undergoes only slight anodic dissolution, producing Ni^2+^, Cu^2+^, and a small amount of Cr^3+^. These ions can react with OH^−^ to form Ni(OH)_2_ and Cu(OH)_2_, which subsequently undergo slow dehydration to form stable oxide films such as NiO, CuO, and Cr_2_O_3_.

Ni − 2e^−^ → Ni^2+^ (11)

Ni^2+^ + 2OH^−^ → Ni(OH)_2_ (12)

Ni(OH)_2_ → NiO + H_2_O (13)

Cu − 2e^−^ → Cu^2+^ (14)

Cu^2+^ + 2OH^−^ → Cu(OH)_2_ (15)

Cu(OH)_2_ → CuO + H_2_O (16)

Cr → Cr^3+^ + 3e^−^ (17)

Cr^3+^ + 3OH^−^ → Cr(OH)_3_↓ (18)

2Cr(OH)_3_ → Cr_2_O_3_ + 3H_2_O (19)

Similarly, owing to the presence of a large amount of Cl^−^, complexation reactions can occur. High concentrations of Cl^−^ can undergo multistep complexation with Ni^2+^, Cu^2+^, and Cr^2+^ dissolved from the binder phase, forming a series of soluble chloride complex ions, including NiCl_m_^(2−m)^, CuCl_m_^(2−m)^, and CrCl_m_^(3−m)^. These complexation reactions consume metal ions at the interface, suppress the precipitation of Ni(OH)_2_, Cu(OH)_2_, and Cr(OH)_3_, and hinder the self-healing of defects in the corrosion-product film and passive film.

### 3.3. Pure Wear Under Cathodic Protection Condition

**Figure 8:**
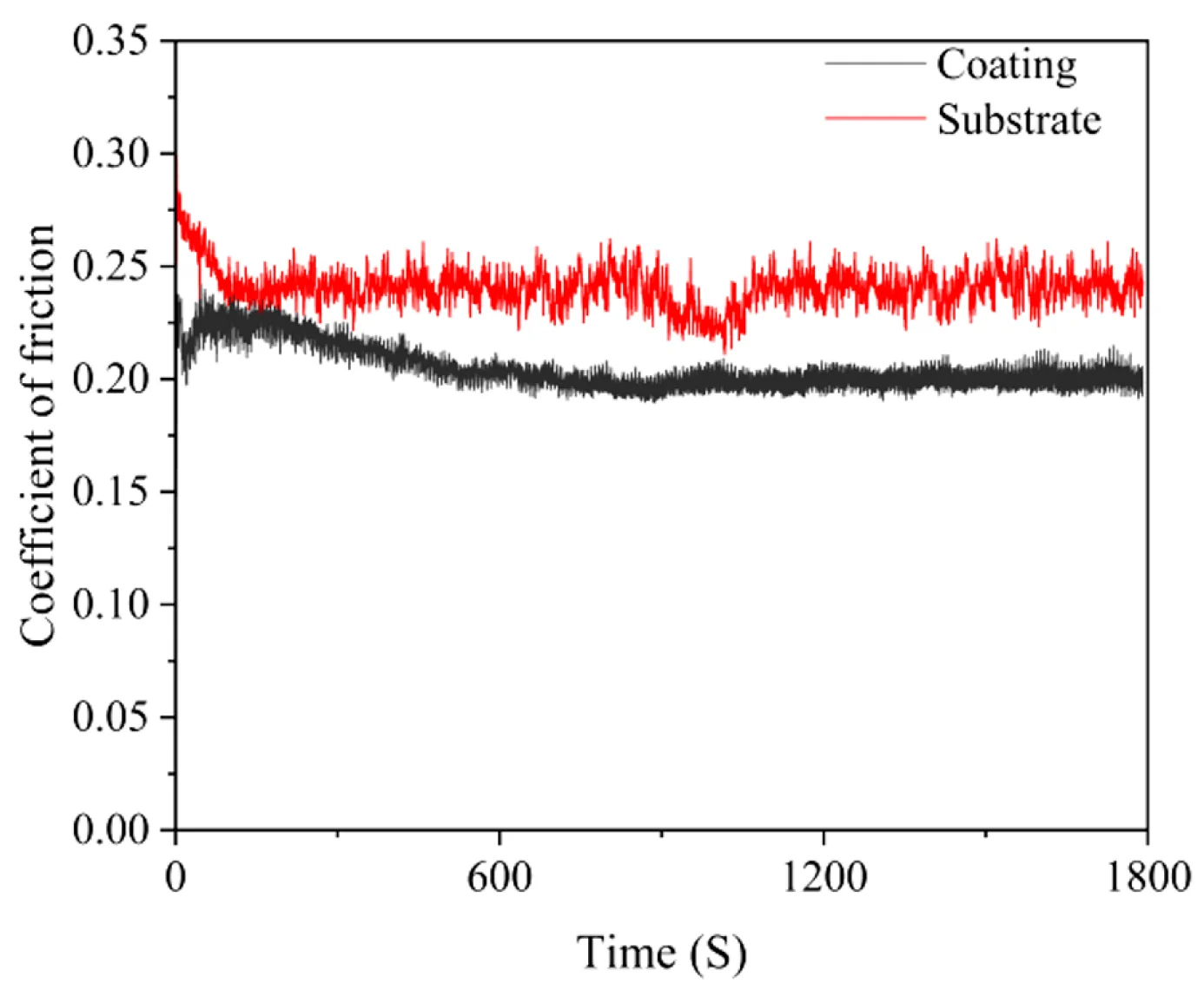
Friction coefficients of specimens under cathodic protection conditions.

To quantitatively determine the material loss of the P550 substrate and the WC/Ni-based coating caused solely by mechanical wear in the drilling fluid, tribological tests were conducted under cathodic protection. By suppressing electrochemical corrosion, the contribution of mechanical wear was quantified independently. Figure 8 shows the friction coefficient of the substrate and the coating under cathodic protection. The friction behavior of both materials can be divided into two stages: a running-in stage and a steady-state friction stage. After a running-in period of approximately 200 s, the friction coefficients exhibited relatively stable fluctuations, indicating that the friction process remained generally stable under cathodic protection. During the steady-state stage, the average friction coefficients of the substrate and the coating were 0.241 ± 0.021 and 0.205 ± 0.016, respectively.

**Figure 9:**
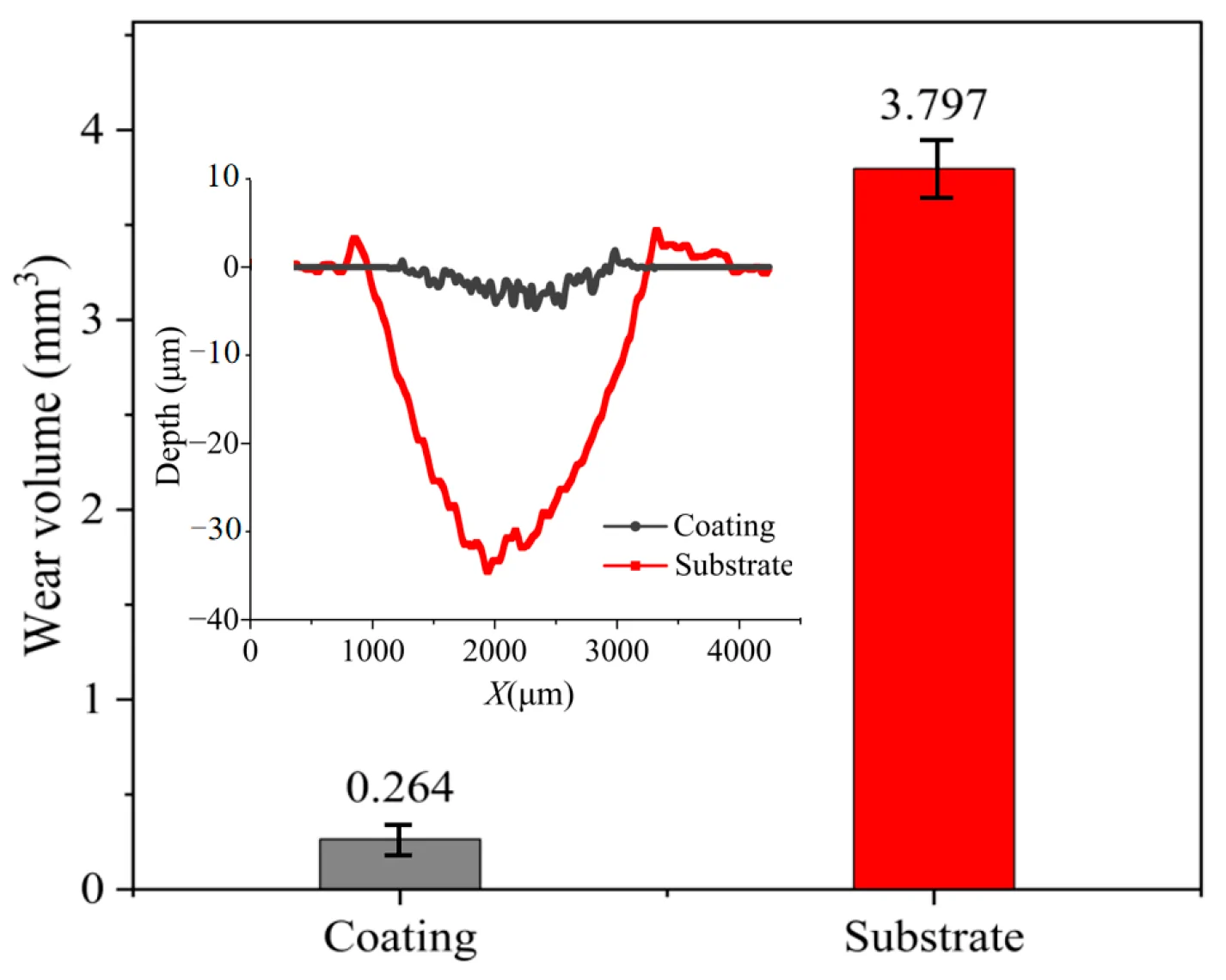
Wear tracks and wear volumes of specimens under cathodic protection conditions.

Figure 9 presents the cross-sectional profiles of the wear tracks and the corresponding wear volume losses of the two materials after testing. It can be observed that the wear depth of the coating is very shallow, while that of the substrate was substantially larger. Under cathodic protection, the wear volume losses of the substrate and the coating were 3.797 ±0.192 mm^3^ and 0.246 ± 0.018 mm^3^, respectively. The wear volume of the coating was significantly lower than that of the substrate. This superior wear resistance is mainly attributed to the WC particle skeleton and hard phases such as W_2_C and Cr_7_C_3_ in the coating. It should be noted that the applied cathodic potential (OCP −1.0 V) is slightly negative to the equilibrium potential of water reduction at pH = 9, so mild hydrogen evolution is thermodynamically possible. However, hydrogen evolution itself does not cause metallic dissolution. Within the 30 min tribological test in weak alkaline drilling fluid, hydrogen atoms only penetrate into an extremely thin near-surface layer, and the resulting extra wear increment is limited. Therefore, the measured wear volume can represent pure mechanical wear for the purpose of decoupling corrosion and wear.

**Figure 10:**
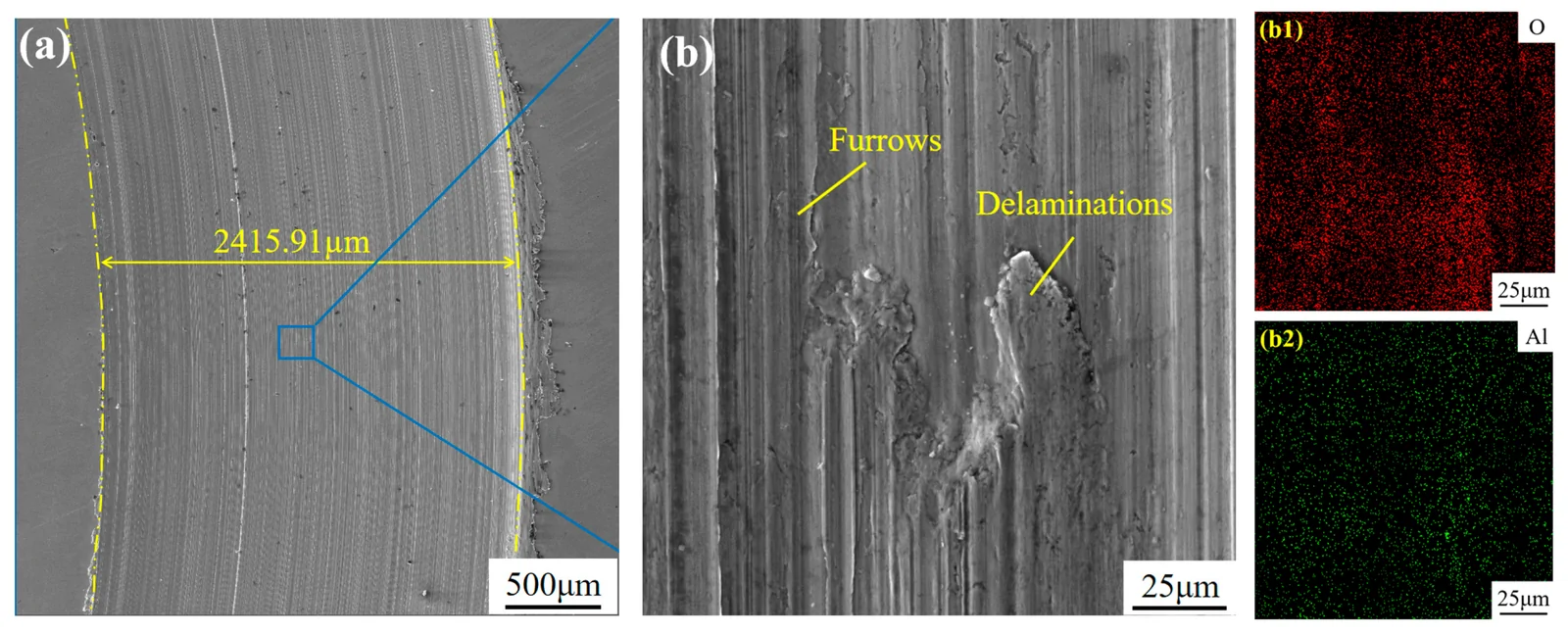
Wear morphologies of substrate under cathodic protection conditions: (**a**) Wear surface; (**b**) Enlarged view; (**b1**) O element mapping; (**b2**) Al element mapping.

**Figure 11:**
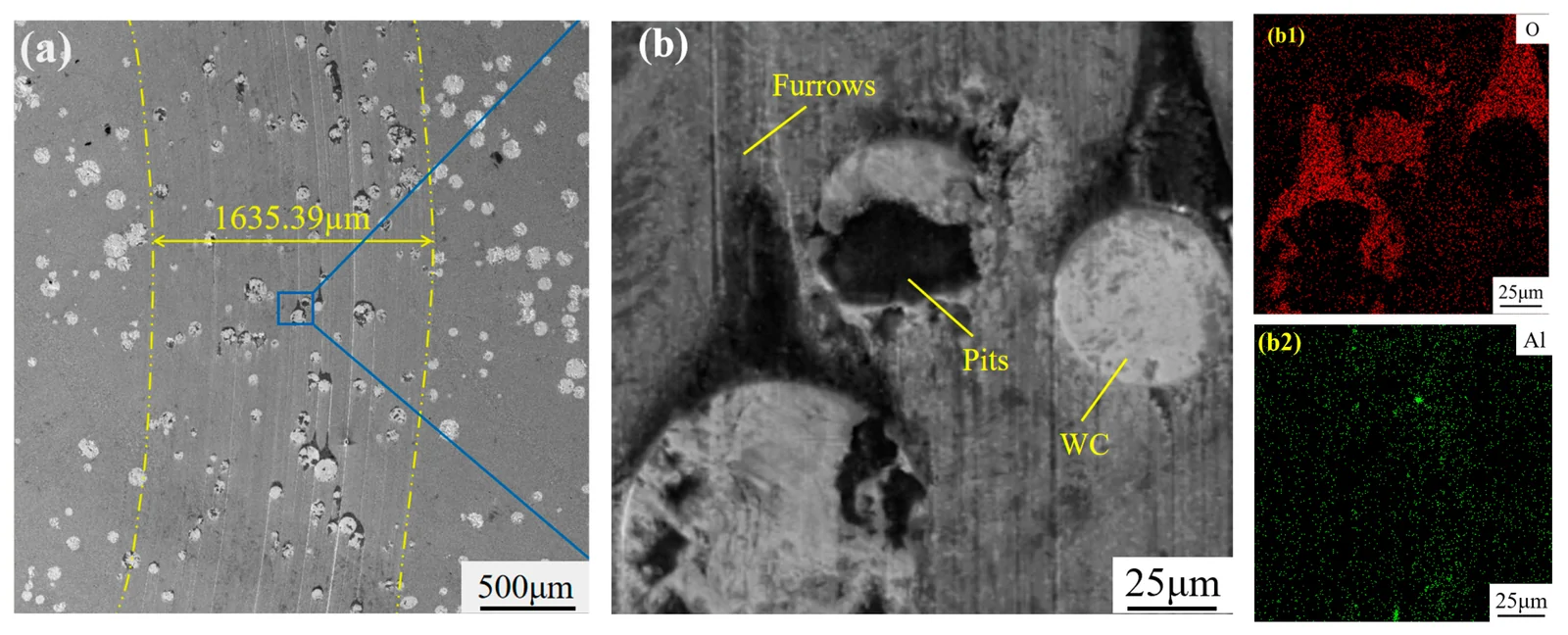
Wear morphologies of coating under cathodic protection conditions: (**a**) Wear surface; (**b**) Enlarged view; (**b1**) O element mapping; (**b2**) Al element mapping.

Figure 10 and Figure 11 show the wear morphologies and EDS mapping results of the substrate and coating under cathodic protection conditions. As shown in Figure 10a,b, the wear track of the substrate under the synergistic action of corrosion and friction is wide and deep. A large number of grooves and plastic deformation appear on the worn surface, accompanied by abrasive particles and delamination, which is mainly characterized by abrasive wear and adhesive wear. In contrast, as shown in Figure 11a,b, the wear track of the coating is narrow, shallow and discontinuous, and no obvious plastic bulging is observed at both edges of the wear track. This is mainly because the large amount of hard WC particles in the coating provides pronounced skeletal support during friction. The WC particles bear most of the cutting load, hinder the plowing action, and effectively suppress surface plastic deformation. However, scratches and material loss are still observed on the WC particles themselves, and a small number of WC particles undergo spalling. The dominant wear mechanism of the coating is abrasive wear.

In addition, the elemental mappings in Figure 10(b1,b2) and Figure 11(b1,b2) reveal the presence of O and Al in the wear regions of both the substrate and the coating. This is attributed to the transfer and adhesion of Al and O from the Al_2_O_3_ counterpart pin during sliding. Slight oxidation during the friction process may be another source of the detected O.

### 3.4. Corrosion–Wear Under Dynamic Polarization Condition

**Figure 12:**
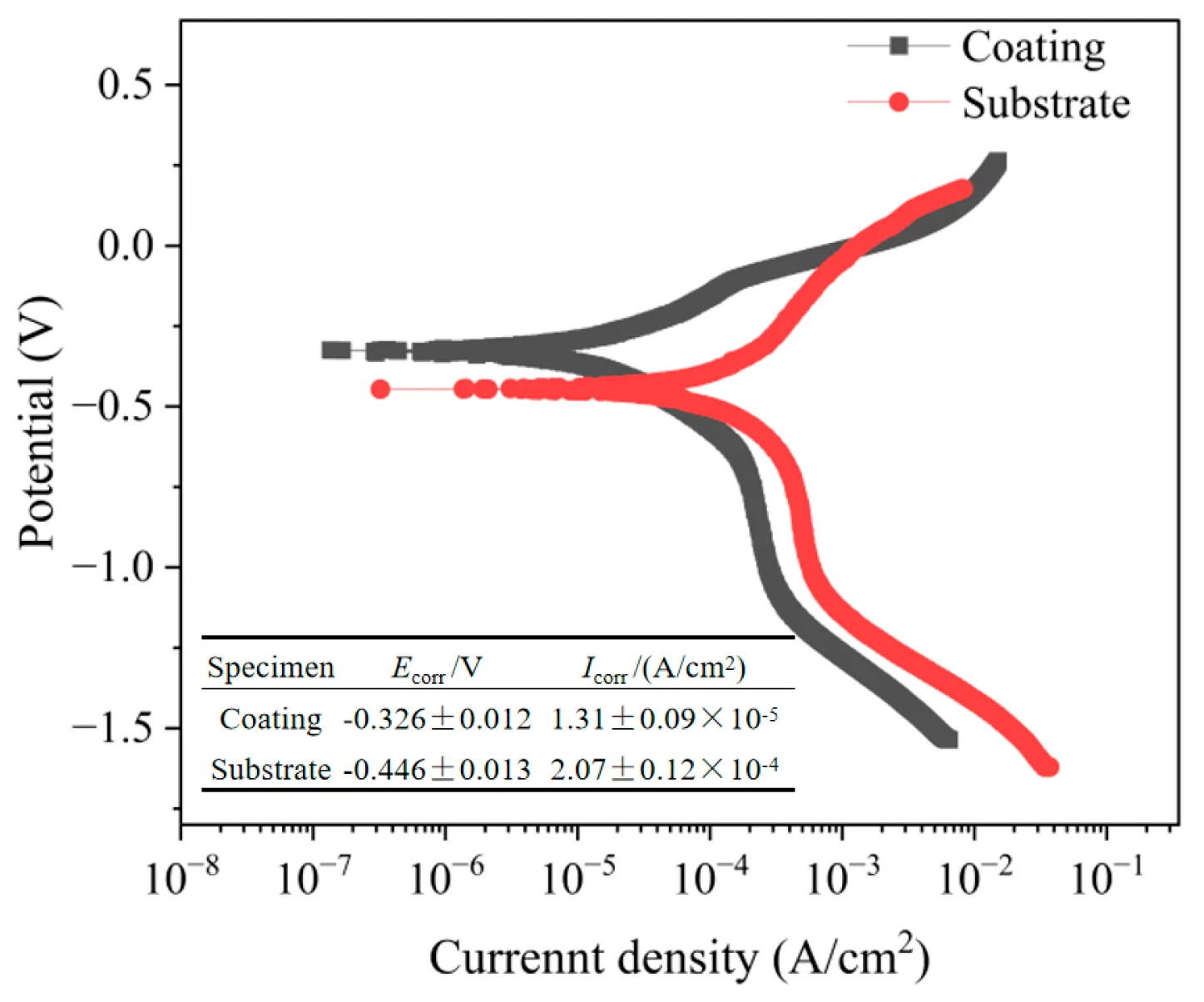
Dynamic polarization curves of specimens under dynamic polarization conditions.

To quantitatively evaluate the corrosion–wear material loss of the P550 substrate and the WC/Ni-based coating in the drilling fluid, corrosion–wear tests were conducted under dynamic polarization condition, in which the materials were simultaneously subjected to electrochemical corrosion and sliding friction. Figure 12 shows the test results, including the potentiodynamic polarization curves and electrochemical parameters of the specimens. Under dynamic polarization condition, the corrosion current density (*I*_corr_) and corrosion potential (*E*_corr_) of the coating were 1.31 ± 0.09 × 10^−5^ A/cm^2^ and −0.326 ± 0.012 V, whereas those of the substrate were 2.07 ± 0.12 × 10^−4^ A/cm^2^ and −0.446 ± 0.013 V, respectively. The coating therefore exhibited a more positive corrosion potential and a lower corrosion current density, indicating superior corrosion resistance.

Compared with the static polarization results obtained in Figure 6a, both the substrate and the coating exhibit a decreased *E*_corr_ and a significantly increased *I*_corr_ under corrosive wear. This indicates that friction accelerates active dissolution at the material surface and promotes the migration and diffusion of ions in the corrosive medium, thereby further intensifying the corrosion process.

**Figure 13:**
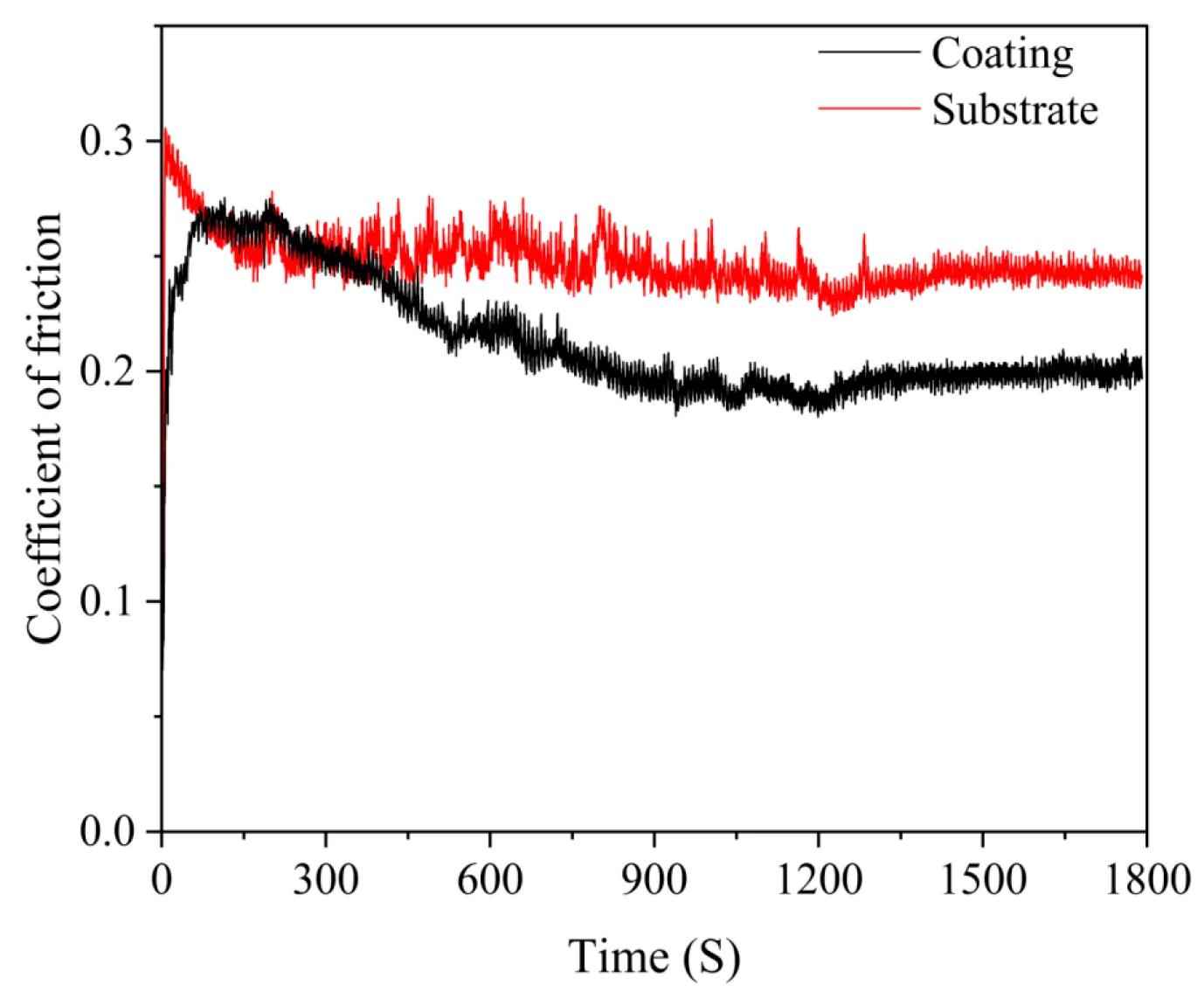
Friction coefficients of specimens under dynamic polarization condition.

The friction coefficient curves of the P550 substrate and the WC/Ni-based coating under dynamic polarization conditions are shown in Figure 13. Compared with the friction coefficient curve under pure wear condition (Figure 8), the substrate also enters a stable friction stage after approximately 200 s. In contrast, the coating reaches the stable wear stage at around 600 s with substantial fluctuations in the running-in period. This phenomenon may be attributed to the considerable material transfer of the Ni-Cu binder phase in the coating under the coupled electrochemistry–friction effect, which creates height differences relative to the WC particle skeleton. Such differences induce inhomogeneous interfacial contact between the counterpart pin and the scratched coating surface, leading to fluctuations. After 600 s, with the continued action of friction and electrochemical corrosion, corrosion products on the material surface were gradually scraped off, and the friction pair entered a state characterized by small-amplitude fluctuations. After 1200 s, the surface asperities were continuously sheared and compacted, leading to a more stable friction process accompanied by a slight increase in the friction coefficient. At this stage, the average friction coefficient of the substrate and coating was 0.240 ± 0.023 and 0.199 ± 0.018, respectively.

**Figure 14:**
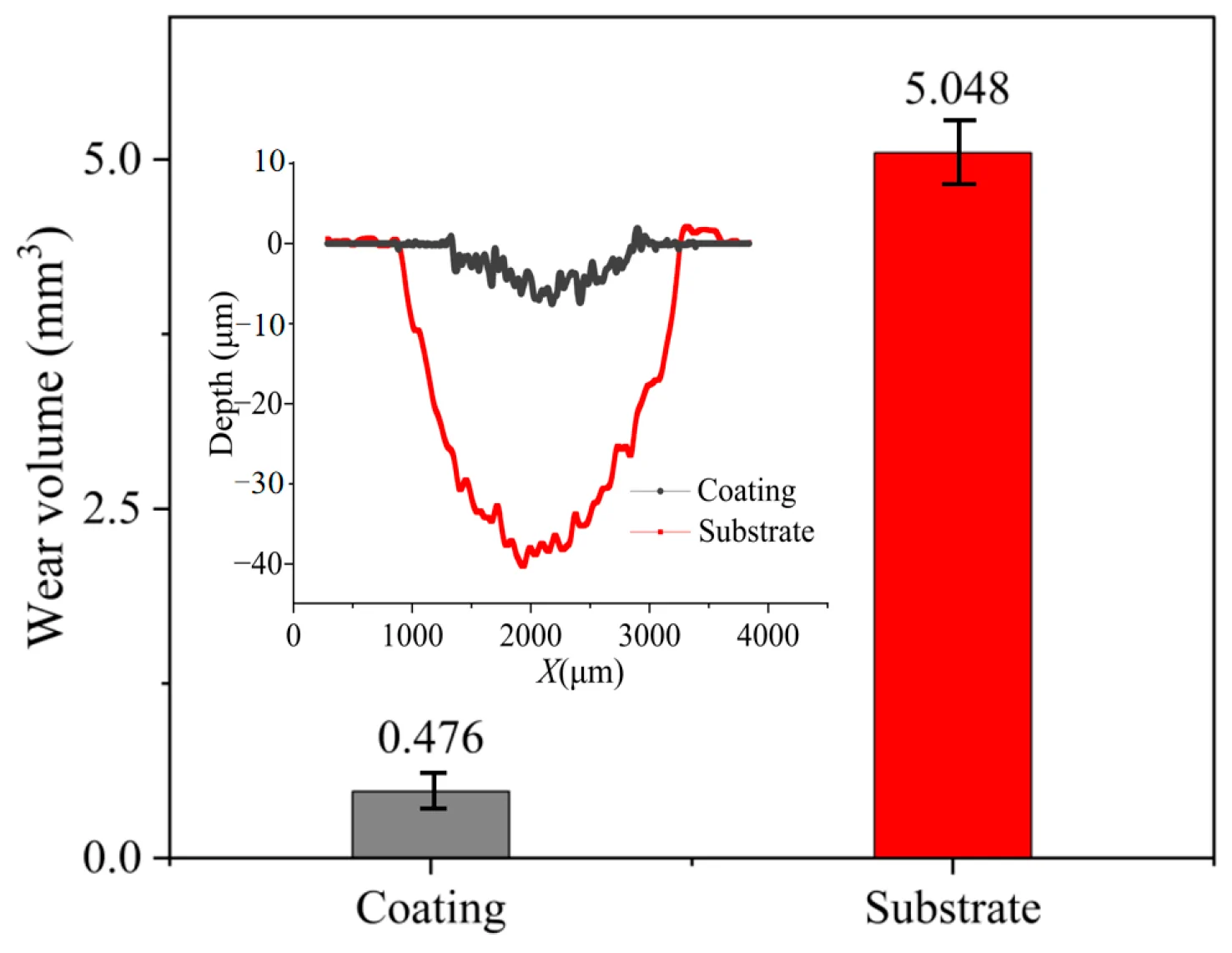
Wear tracks and wear volumes of specimens under dynamic polarization conditions.

The cross-sectional profiles of the wear tracks and the corresponding wear volume losses of the specimens are shown in Figure 14. Under corrosion–wear conditions, the wear depth of the coating was much shallower than that of the substrate. The wear volumes of the substrate and coating were 5.048 ± 0.249 mm^3^ and 0.476 ± 0.033 mm^3^, respectively. Compared with those obtained under the pure wear condition (Figure 9), the wear volumes increased markedly.

**Figure 15:**
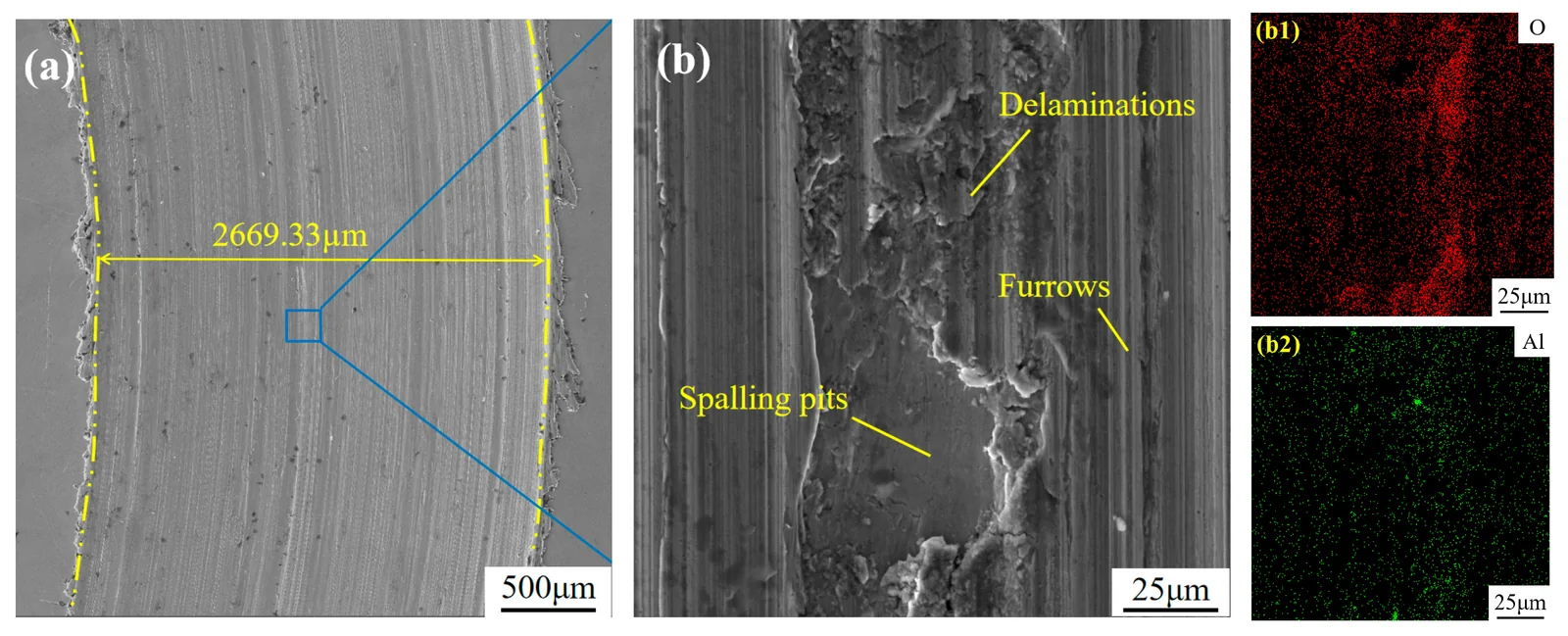
Wear morphologies of the substrate under dynamic polarization conditions: (**a**) Wear surface; (**b**) Enlarged view; (**b1**) O element mapping; (**b2**) Al element mapping.

**Figure 16:**
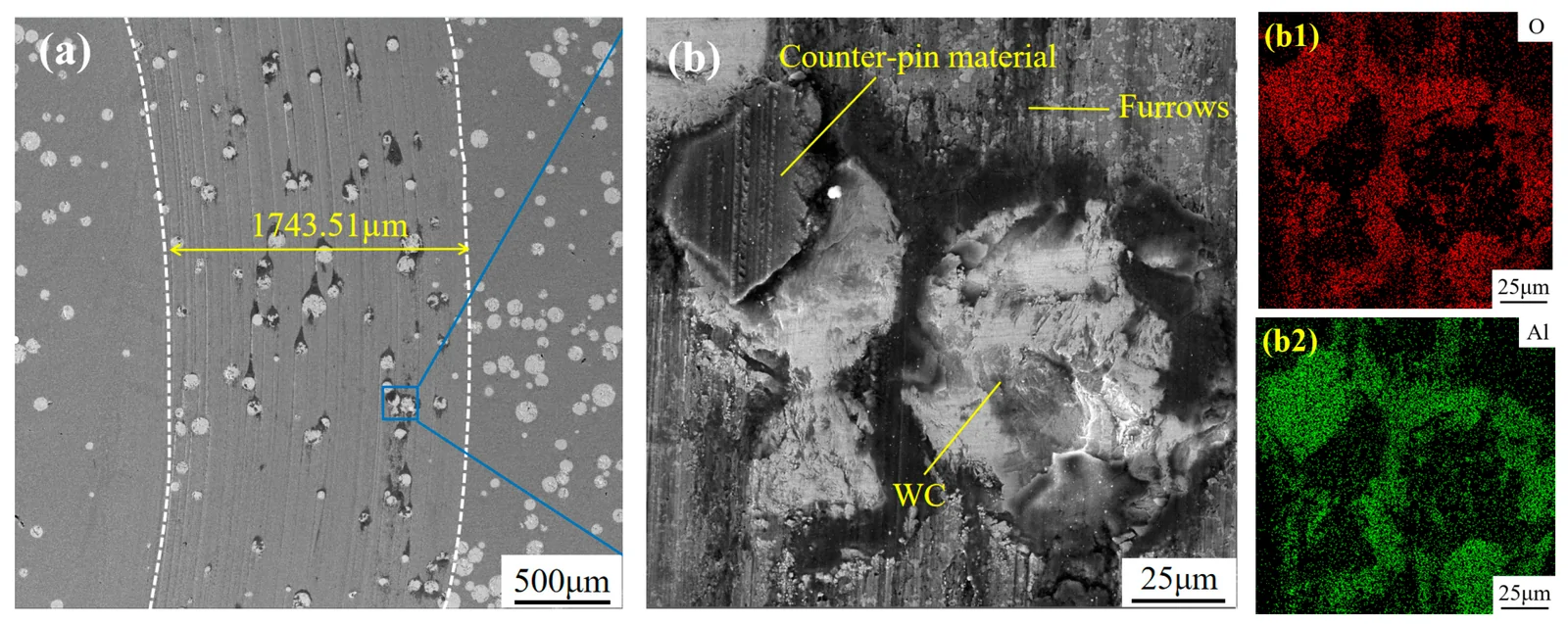
Wear morphologies of the coating under dynamic polarization conditions: (**a**) Wear surface; (**b**) Enlarged view; (**b1**) O element mapping; (**b2**) Al element mapping.

The surface morphologies of the substrate and coating after corrosion–wear testing are shown in Figure 15 and Figure 16. Compared with the pure wear under cathodic protection conditions shown (Figure 10 and Figure 11), the surface wear damage of the substrate and the coating was significantly aggravated under dynamic polarization. The wear tracks became wider and the plowing grooves deeper. This is because, during the corrosion–wear process, electrochemical corrosion degrades the surface properties of the material and weakens the bonding strength of the surface layer, further exacerbating surface damage of the material. Among them, numerous furrows, tearing-induced delamination and spalling pits are observed on the wear surface of the P550 substrate (Figure 15b). In Figure 16b, on the wear surface of the coating, it can be seen that WC particles undergo fragmentation under the coupled action of corrosion and wear. Meanwhile, owing to the blocking and shearing effects of the WC particles, Al_2_O_3_ is scraped by the WC particles and leaves residual debris in the coating. As can be seen from Figure 15(b1,b2) and Figure 16(b1,b2), substantial amounts of Al and O originating from the counterpart pin remain on the surfaces of both the substrate and coating.

In general, whether under pure wear or corrosive-wear condition, the coating exhibits far less damage than the P550 substrate. On the one hand, the overall hardness of the coating is markedly higher than that of the substrate, endowing it with superior resistance to mechanical extrusion and wear deformation. On the other hand, WC particles, together with W_2_C and Cr_2_C phases, are uniformly and dispersedly distributed inside the coating and serve as the core wear-resistant reinforcing phases. These hard phases bear the main interfacial friction load and block the direct impact of frictional stress on the inner substrate during friction. Meanwhile, WC particles possess excellent corrosion resistance, and the abundant dispersed WC particles reduce the corrosion rate of the material surface.

### 3.5. Corrosion–Wear Interaction Analysis

According to the classical damage decomposition theory of corrosion–wear, the total material volume loss can be divided into three components: the volume loss due to pure corrosion, the volume loss due to pure mechanical wear, and the additional volume loss arising from the interaction between corrosion and wear [24]:

(20) $$V_{\mathrm{total}}=V_{\mathrm{corr}}^{\prime }+V_{\mathrm{wear}}+\Delta V$$

where *V*_total_ is the total volume loss under corrosion–wear conditions, $V_{\mathrm{corr}}^{\prime }$ is the volume loss caused by pure corrosion under static corrosion conditions, *V*_wear_ is the volume loss caused by pure wear under cathodic protection, and Δ*V* is the additional volume loss resulting from the interaction between corrosion and wear. Δ*V* can be further divided into two components:

(21) $$\Delta V=\Delta V_{c}^{\prime }+\Delta V_{w}$$

with $\Delta V_{c}^{\prime }$ being the volume loss arising from wear-accelerated corrosion and Δ*V*_w_ being the volume loss caused by corrosion-promoted wear.

Accordingly, the total volume loss under corrosion–wear conditions can be further expressed as follows:

(22) $$V_{\mathrm{total}}=V_{\mathrm{corr}}^{\prime }+V_{\mathrm{wear}}+\Delta V_{c}^{\prime }+\Delta V_{w}$$

To further distinguish the respective contributions of corrosion and wear during the corrosion–wear process, the total corrosion-induced volume loss $V_{C}^{\prime }$ and the total wear-induced volume loss *V*_W_ can be defined as follows:

(23) $$V_{C}^{\prime }=V_{\mathrm{corr}}^{\prime }+\Delta V_{c}^{\prime }$$

*V*_W_ = *V*_wear_ + Δ*V*_w_ (24)

Therefore, $\Delta V_{c}^{\prime }$ and Δ*V*_w_ can be calculated using the following equations:

(25) $$\Delta V_{c}^{\prime }=V_{c}^{\prime }-V_{\mathrm{corr}}^{\prime }$$

(26) $$\Delta V_{w}=V_{\mathrm{total}}-V_{\mathrm{corr}}^{\prime }-V_{\mathrm{wear}}-\Delta V_{c}^{\prime }$$

**Figure 17:**
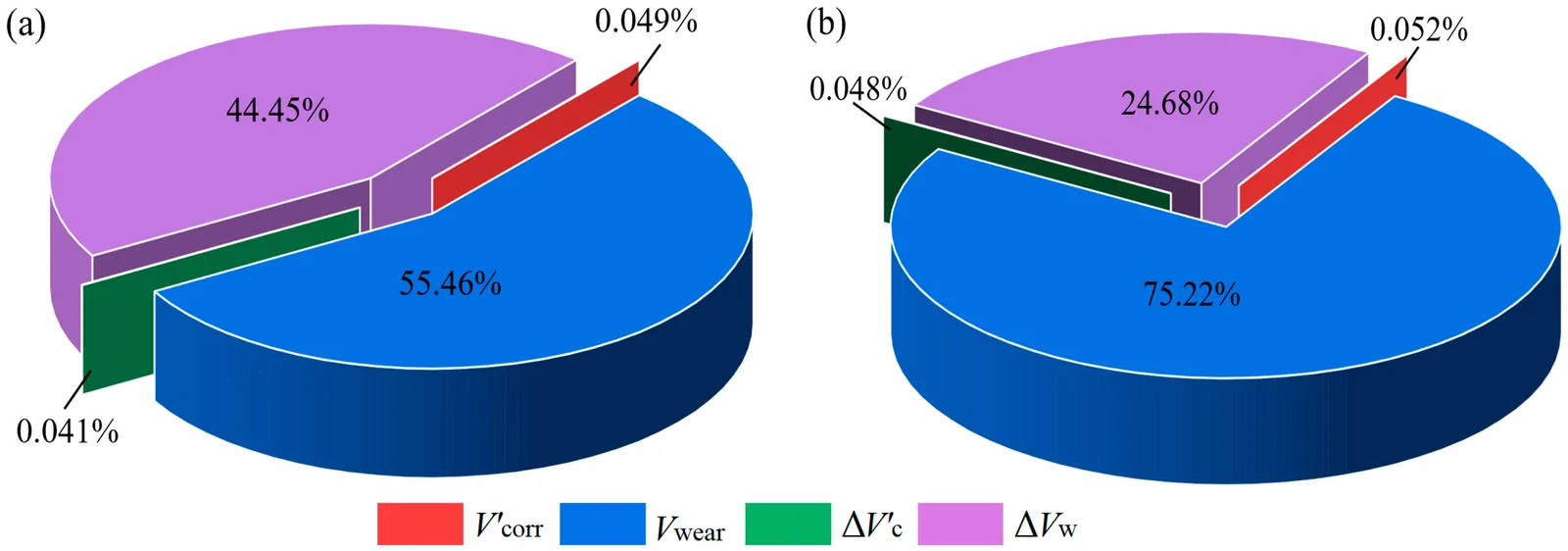
Volume loss proportions of each component: (**a**) Coating. (**b**) Substrate.

Based on the measurement results, the total volume loss under corrosion–wear condition, the volume loss due to pure corrosion, the volume loss due to pure wear, and the additional volume loss caused by the corrosion–wear interaction were calculated using Equations (1) and (2). All data are listed in Table 4. In addition, the fractions of each volume loss component in the total volume loss are presented in Figure 17.

As shown in Table 4 and Figure 17, in general, the total volume loss of the coating is 0.476 mm^3^, which is markedly lower than the 5.048 mm^3^ for the substrate, indicating that the coating possesses outstanding corrosion–wear resistance. Further analysis shows that, for both the substrate and the coating, the total corrosion-induced volume loss $V_{c}^{\prime }$ is very small, whereas the total wear-induced volume loss *V*_w_ is very big. This indicates that material degradation is governed primarily by wear-related processes.

With respect to the individual volume loss components, there is a significant difference between the coating and the substrate during the corrosion–wear process. For the coating, the pure corrosion volume loss $V_{\mathrm{corr}}^{\prime }$ is 2.33 × 10^−4^ mm^3^, accounting for 0.049% of the total volume loss, while the additional volume loss caused by wear-accelerated corrosion $\Delta V_{c}^{\prime }$ is 1.93 × 10^−4^ mm^3^, accounting for 0.041%. The promotion effect of wear on corrosion volume loss is nearly doubled. In contrast, the pure mechanical wear volume loss *V*_wear_ is 0.264 mm^3^, accounting for 55.46%, whereas the additional volume loss caused by corrosion-accelerated wear Δ*V*_w_ is 0.211574 mm^3^, accounting for as much as 44.45%. It is evident that corrosion dramatically accelerates the wear volume loss.

For the substrate, the pure corrosion volume loss $V_{\mathrm{corr}}^{\prime }$ is 2.63 × 10^−3^ mm^3^, accounting for 0.052% of the total volume loss, and the additional volume loss induced by wear-accelerated corrosion is nearly equivalent. In contrast, the pure mechanical wear volume loss *V*_wear_ is 3.797 mm^3^, accounting for 75.22%, whereas the additional volume loss caused by corrosion-accelerated wear Δ*V*_w_ is 1.2459 mm^3^, accounting for 24.68%. It indicates that pure mechanical wear is the primary damage mode and corrosion greatly accelerates wear.

Furthermore, in the corrosion–wear system consisting of the substrate and the coating, the effect of corrosion-accelerated wear is significantly stronger than that of wear-accelerated corrosion, showing obvious asymmetry in the interaction. The main reason is that the corrosive medium does not greatly increase the direct dissolution loss of materials. Instead, it damages the structural stability of the surface layer, reducing the local load-bearing capacity and interfacial bonding strength. Consequently, the material is more prone to particle detachment, microcrack propagation and local spalling under frictional load, which leads to a remarkable increase in volumetric wear loss.

Compared with the substrate, the coating exhibits a higher volume loss proportion induced by corrosion-accelerated wear, and the synergistic corrosion–wear damage is more prominent. The corrosive medium can penetrate into coating defects such as pores and intergranular boundaries, inducing localized corrosion at these weak sites. The formed corrosion products with low hardness cannot provide effective lubrication, and they are readily stripped away by the reciprocating sliding contact, further exposing fresh reactive surfaces and amplifying the material removal. Therefore, subsequent coating performance optimization should focus on improving the structural integrity of the coating surface layer and the resistance to corrosion-induced spalling, so as to effectively suppress the acceleration effect of corrosion on wear.

It is worth noting that the above corrosion–wear synergy was characterized under potentiodynamic scanning coupled with sliding, rather than at a fixed potential or OCP. Potentiodynamic polarization was coupled with sliding friction to investigate corrosion–wear responses across a broad potential range, mimicking the dynamically fluctuating potential of downhole components in drilling fluid. It should be noted that the continuously varied potential history during scanning modulates the interfacial electrochemical reactions, film formation and destruction in real time. Consequently, the obtained corrosion–wear synergy represents an integrated result over the scanned potential window, which is different from the synergy measured at a fixed potential or OCP under steady-state electrochemistry.

### 3.6. Corrosion–Wear Promotion Mechanism

**Figure 18:**
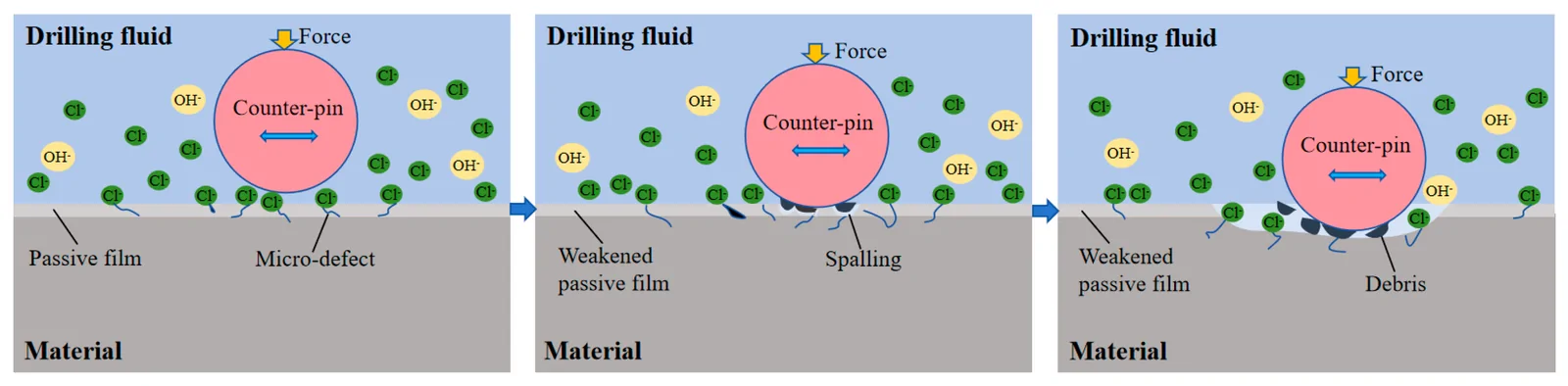
Schematic diagram of corrosion-promoted wear mechanism.

The core mechanism by which corrosion promotes wear is the corrosion-induced weakening of the material surface, which in turn aggravates wear, as shown in Figure 18. The saturated brine drilling fluid contains a small amount of OH^−^ and a large concentration of highly active Cl^−^. First, Cl^−^ preferentially adsorbs at defects in the passive film on the material surface and penetrates into the subsurface region through surface microdefects and pores. They displace oxygen atoms within the film and combine with cations to form soluble salts, thereby destroying the structural integrity and continuity of the passive film. This process not only weakens the bonding strength between the passive film and the material, but also generates stress-concentration sites such as pitting pits and microcracks on the surface, leading to significant deterioration in the mechanical properties of the near-surface material. And then, under the continuous frictional loading imposed by the counterpart, the load-bearing capacity of the corrosion-weakened passive film is markedly reduced, and the damaged surface layer becomes highly susceptible to plastic deformation, tearing and detachment of surface particles. At last, the detached corrosion products and debris remain trapped within the frictional contact interface and act as third-body abrasives, continuously cutting, crushing and scratching the material surface, thereby further amplifying the mechanical wear effect.

**Figure 19:**
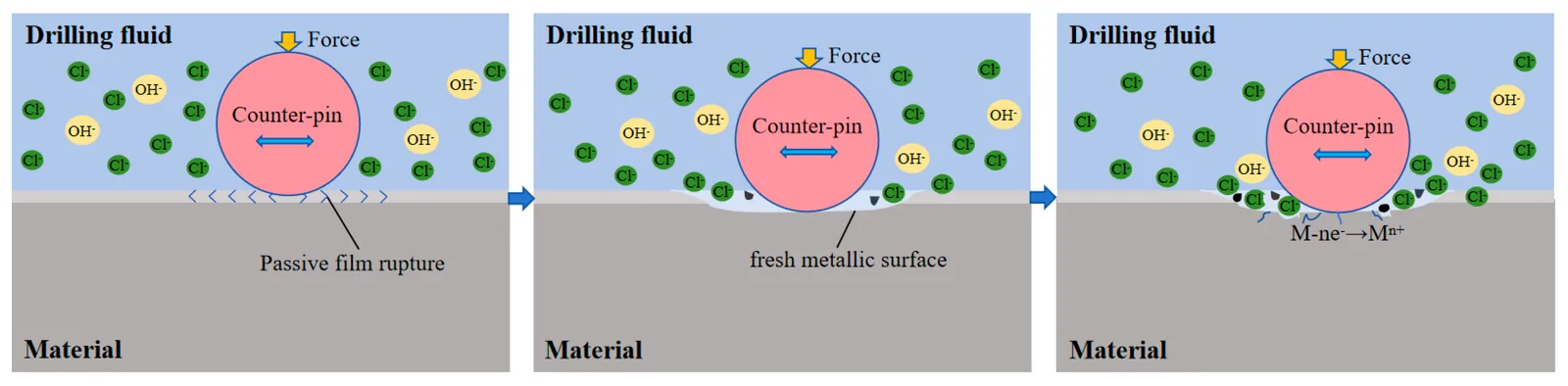
Schematic diagram of wear-promoted corrosion mechanism.

The core mechanism by which corrosion promotes wear is the mechanical disruption of the electrochemical stability of the material surface, which significantly accelerates anodic dissolution, as shown in Figure 19. First, periodic sliding friction disrupts the electrochemical stability of the material surface, resulting in scratching, rolling and tearing of the passive film. Subsequently, under continuous plowing and cutting actions of friction force, the passive film on the material surface detaches, and the highly reactive fresh metallic surface is directly exposed to the aggressive corrosive medium, leading to a negative shift in the open-circuit potential. Thereafter, the exposed fresh metallic surface exhibits markedly higher electrode activity than the passive-film-covered regions, which forms galvanic corrosion with the unworn zones and greatly accelerates the anodic dissolution of active metallic constituents. The generated metal cations M^n+^ further react with anions such as Cl^−^ and OH^−^ in the medium to form soluble chloride complex salts, continuously triggering anodic dissolution corrosion.

## 4. Conclusions

In this paper, P550 nonmagnetic stainless steel and laser-clad WC/Ni-based composite coating were selected as research objects. A series of independent corrosion, independent wear and coupled corrosion–wear tests were carried out systematically in saturated brine drilling fluid. The electrochemical behavior, tribological response and micro-damage characteristics of materials were analyzed, and the interaction mechanism between corrosion and wear was revealed. The main conclusions are as follows:

(1) Under static pure corrosion conditions, the WC/Ni-based coating exhibits a more positive corrosion potential and lower corrosion current density, with markedly better corrosion resistance than the P550 nonmagnetic steel substrate. The substrate suffers from uniform corrosion, while the composite coating undergoes selective corrosion. Only the Ni-Cu binder phase dissolves locally, and the overall microstructure of the coating remains stable. Under pure wear with cathodic protection, severe plastic deformation, plowing and material spalling occur on the substrate, leading to serious wear damage. The WC/Ni-based coating has a much lower wear volume than the substrate, and its wear mechanism is dominated by mild abrasive wear.
(2) Under coupled corrosion–wear condition, corrosion and wear exhibit a remarkable bidirectional synergistic effect. Mechanical friction continuously destroys the surface passive film and accelerates anodic dissolution, raising the corrosion activity and corrosion current density of the material. Meanwhile, the corrosive medium weakens the surface structure and reduces the interfacial bonding strength of phases, which further aggravates material wear. Severe damage occurs on the substrate under coupled conditions, with coexisting abrasive wear, adhesive wear and oxidative wear. Benefiting from the supporting effect of the WC hard phase skeleton, the coating effectively inhibits plastic deformation and particle detachment and delays the inward penetration of corrosive medium. Its wear mechanism is dominated by adhesive wear. The total volume loss of the coating is far lower than that of the substrate, demonstrating outstanding corrosion–wear resistance.
(3) The corrosion–wear interaction shows obvious asymmetry. Nearly 99% of the total volume loss of both the substrate and the coating originates from mechanical wear. The volume increment induced by corrosion-accelerated wear accounts for 44.45% and 24.68% of the total loss of the substrate and coating, respectively. In contrast, the volume loss caused by wear-accelerated corrosion accounts for merely 0.041% and 0.048%. The acceleration effect of corrosion on wear is absolutely dominant and far exceeds the promotion effect of wear on corrosion. Through surface weakening, internal defect propagation and mechanical property deterioration, corrosion significantly amplifies the volume loss caused by mechanical wear, serving as the core inducement of coupled material damage. The above results reveal that the corrosion–wear interaction is not a symmetric synergistic effect but is dominated by the pathway of corrosion-induced surface weakening followed by wear-mediated damage amplification, which is the core inducement of coupled material degradation.
(4) The laser-clad WC/Ni-based composite reinforced material can significantly enhance the corrosion–wear resistance of nonmagnetic stainless steel in saturated brine drilling fluid and effectively suppress the synergistic damage caused by corrosion and wear, providing a reliable theoretical basis and technical support for surface strengthening and extension of the service life of nonmagnetic drill collars.

## Figures and Tables

**Table 1:** Chemical composition of P550 nonmagnetic steel (wt.%).

C	N	Cr	Mn	Ni	Mo	Si	Fe
≤0.05	0.5~0.6	17.5~19	18.5~20	≤1.5	≤0.8	≤0.5	Bal.

**Table 2:** Chemical composition of nonmagnetic WC/Ni-based powder (wt.%).

WC	C	Cu	Fe	B	Cr	Mn	Ni
60	≤0.05	7.69	0.85	0.45	1.44	1.05	Bal.

**Table 3:** Electrochemical parameters of specimens.

Specimen	*E*_corr_/V	*I*_corr_/(A/cm^2^)	*n*_1_	*R*_p_/(Ω∙cm^2^)	*CPE*_1_/F∙cm^−2^	χ^2^
Coating	−0.266 ± 0.010	2.68 ± 0.13 × 10^−6^	0.87 ± 0.13	35,084 ± 1460	5.71 ± 0.23 × 10^−5^	3.2 ± 0.3 × 10^−4^
Substrate	−0.373 ± 0.013	1.43 ± 0.08 × 10^−5^	0.83 ± 0.13	32,846 ± 1168	5.85 ± 0.21 × 10^−5^	4.2 ± 0.3 × 10^−4^

**Table 4:** Volume loss of each component in the specimens.

Specimen	*V*_total_/mm^3^	$V_{C}^{\prime }$/mm^3^	*V*_W_/mm^3^	$V_{\mathrm{corr}}^{\prime }$/mm^3^	*V*_wear_/mm^3^	Δ*V*/mm^3^	
						$\Delta V_{c}^{\prime }$/mm^3^	Δ*V*_w_/mm^3^
Coating	0.476	4.26 × 10^−4^	0.475574	2.33 × 10^−4^	0.264	1.93 × 10^−4^	0.211574
Substrate	5.048	5.07 × 10^−3^	5.0429	2.63 × 10^−3^	3.797	2.44 × 10^−3^	1.2459

## Data Availability

The original contributions presented in this study are included in the article. Further inquiries can be directed to the corresponding author.
